# Increased TRPV4 Channel Expression Enhances and Impairs Blood Vessel Function in Hypertension

**DOI:** 10.1161/HYPERTENSIONAHA.124.23092

**Published:** 2024-10-23

**Authors:** Xun Zhang, Charlotte Buckley, Matthew D. Lee, Christine Salaun, Margaret MacDonald, Calum Wilson, John G. McCarron

**Affiliations:** Strathclyde Institute of Pharmacy and Biomedical Sciences, University of Strathclyde, Glasgow, United Kingdom.

**Keywords:** endothelial cells, hypertension, inositol 1,4,5-trisphosphate receptors, TRPV cation channels, vasodilation

## Abstract

**BACKGROUND:**

Endothelial cell TRPV4 (transient receptor potential vanilloid 4) channels provide a control point that is pivotal in regulating blood vessel diameter by mediating the Ca^2+^-dependent release of endothelial-derived vasoactive factors. In hypertension, TRPV4-mediated control of vascular function is disrupted, but the underlying mechanisms and precise physiological consequences remain controversial.

**METHODS:**

Here, using a comprehensive array of methodologies, endothelial TRPV4 channel function was examined in intact mesenteric resistance arteries from normotensive Wistar-Kyoto and spontaneously hypertensive rats.

**RESULTS:**

Our results show there is a notable shift in vascular reactivity in hypertension characterized by enhanced endothelium-dependent vasodilation at low levels of TRPV4 channel activation. However, at higher levels of TRPV4 activity, this vasodilatory response is reversed, contributing to the aberrant vascular tone observed in hypertension. The change in response, from dilation to constriction, was accompanied by a shift in intracellular Ca^2+^ signaling modalities arising from TRPV4 activity. Oscillatory TRPV4-evoked IP_3_ (inositol triphosphate)-mediated Ca^2+^ release, which underlies dilation, decreased, while the contraction inducing sustained Ca^2+^ rise, arising from TRPV4-mediated Ca^2+^ influx, increased. Our findings also reveal that while the sensitivity of endothelial cell TRPV4 to activation was unchanged, expression of the channel is upregulated and IP_3_ receptors are downregulated in hypertension.

**CONCLUSIONS:**

These data highlight the intricate interplay between endothelial TRPV4 channel expression, intracellular Ca^2+^ signaling dynamics, and vascular reactivity. Moreover, the data support a new unifying hypothesis for the vascular impairment that accompanies hypertension. Specifically, endothelial cell TRPV4 channels play a dual role in modulating blood vessel function in hypertension.

**NOVELTY AND RELEVANCE**

**What Is New?**

In normotension, endothelial TRPV4 (transient receptor potential vanilloid 4) induces vasodilation regardless of the degree of channel activation.

In hypertension, TRPV4 has a dual effect: the channel evokes vasodilation with mild activation but triggers contraction when strongly activated.

**What Is Relevant?**

In hypertension there is a shift in response, from dilation to constriction, brought about by changes in the temporal features of Ca^2+^ signaling mediated by altered expression of TRPV4 and IP_3_ (inositol triphosphate) receptors.

**Clinical/Pathophysiological Implications?**

TRPV4 channels may serve as a compensatory mechanism to counteract rising blood pressure and contribute to the elevated blood pressure that occurs in hypertension.

**See Editorial, pp 69-71**

Hypertension is an insidious condition linked to various vascular disorders, including coronary artery disease, stroke, dementia, and renal failure. Although increased blood pressure levels are positively and continuously related to increasing cardiovascular risk, the precise mechanisms that lead from hypertension to ill health are poorly understood. Nonetheless, changes in arterial structure and function, triggered by endothelial cell dysfunction,^1^ are central to hypertension onset and progression.^2–4^

The endothelial cell lining of blood vessels controls most cardiovascular functions. For example, the endothelium controls the regulation of inflammation, vascular remodeling,^5,6^ and the moment-to-moment control of blood flow.^7,8^ The endothelium regulates each vascular functionality by releasing various diffusible vasoactive molecules. These substances include anti-inflammatory and vasodilator factors (eg, nitric oxide, prostacyclin, and endothelium-derived hyperpolarizing factor) and proinflammatory/vasoconstrictor factors (eg, reactive oxygen species, prostanoids, and endothelin). Often, pathological conditions are associated with a shift from an anti-inflammatory to proinflammatory state, leading to vascular inflammation, narrowed blood vessels, and reduced blood flow. Whether a cause or a consequence, these alterations contribute substantially to the progression of hypertension.^9,10^ However, whether the changes result from a redirection of existing signaling pathways or recruitment of new ones remains uncertain.

The release of endothelium-derived relaxing and contracting factors is triggered by changes in cytosolic Ca^2+^ concentration.^11–14^ The regulation of endothelial Ca^2+^ involves both internal and extracellular sources.^15^ Primarily, Ca^2+^ release from the internal store is mediated by IP_3_ (inositol triphosphate) receptors,^16^ and several Ca^2+^ channels expressed on the plasmalemma membrane control Ca^2+^ influx. Among these influx channels, the TRPV4 (transient receptor potential vanilloid 4) is now recognized as having particular significance in regulating Ca^2+^ entry in endothelial cells.^17–19^ Widely expressed in vascular endothelial cells, TRPV4 channels exhibit high Ca^2+^ permeability and respond to physical stimuli like shear stress, stretch, and intravascular pressure to promote endothelium-dependent vasodilation.^14,20,21^

In hypertension, the potential importance of TRPV4 to vascular control is increased since the physical forces that act on the endothelium (such as shear stress and intravascular pressure) are substantially altered. The modified physical forces raise the possibility that altered TRPV4-mediated Ca^2+^ influx may be a component of the endothelial changes that are associated with hypertension. In support, in a nitric oxide synthase inhibitor-induced model of hypertension, blood pressure was greater in global TRPV4 knockout than in control normotensive mice. The authors proposed that endothelial TRPV4 channel-dependent vasodilation opposes blood pressure increases generated by the nitric oxide synthase inhibitor.^22^

Yet, despite their acknowledged importance to endothelial Ca^2+^ influx, published studies present a complicated picture of the role of endothelial TRPV4 channels in hypertension. While the physical forces that activate TRPV4 are increased, the activity of the channel is reported to be decreased in various models of hypertension.^23–25^ The observations of decreased TRPV4 activity have led to the proposal that strategies to increase endothelial TRPV4 activity may restore vascular function in hypertension. In other studies, TRPV4 activity has been reported to be largely unaltered or even increased in hypertension.^26^ Further confusing the situation, the alterations in TRPV4 activity that occur in hypertension may either increase vasodilation,^26^ reduce vasodilation,^23–25^ evoke endothelium-dependent contractions,^27^ or have little effect.^28^ As a result of the various reported changes in TRPV4 activity, numerous hypotheses have emerged concerning the physiological or pathophysiological implications of altered TRPV4 activity in hypertension. These include the following: TRPV4 may potentially serve no substantial role in the blood pressure changes,^29,30^ may act as a compensatory mechanism to offset elevations in blood pressure,^26^ or may be a cause that underlies the increased blood pressure that characterizes hypertension.^27^

Our study was undertaken to examine endothelial TRPV4-mediated Ca^2+^ responses in hypertension. In particular, we sought to determine whether changes in endothelial function that occur in hypertension were associated with redirection of TRPV4 responses. We show there is increased TRPV4 expression and TRPV4-mediated Ca^2+^ signaling in hypertension. In normotensive controls, TRPV4 activation induced an endothelium-dependent vasodilation at all levels of channel activity. In hypertension, TRPV4 activation generated increased vasodilator responses at low levels of channel activation. However, at higher levels of TRPV4 activation, there was reduced vasodilation, generating an increased contraction. Two features explained the switch in TRPV4-evoked vasomotor responses in hypertension: (1) a reduction in IP_3_-mediated Ca^2+^ signaling and (2) increased TRPV4 Ca^2+^ influx. These results show TRPV4 channels play a dual role in hypertension. Low levels of TRPV4 activity may offer some protection, while at higher levels of activity, TRPV4 may contribute to the increased vascular tone that accompanies hypertension.

## Methods

### Data Availability

All study data are included in the article and supporting information. An expanded Material and Methods section can be found in the Supplemental Materials.

### Animals

Animal care and experimental procedures were conducted in accordance with the relevant UK Home Office Regulations (Schedule 1 of the Animals [Scientific Procedures] Act 1986, United Kingdom) and were approved by the University of Strathclyde Animal Welfare and Ethical Review Body. Animal studies are reported in compliance with the ARRIVE guidelines.^31^

A total of 42 Wistar-Kyoto (WKY, 7 weeks old) and 42 spontaneously hypertensive (SHR, 8 weeks old) rats (purchased from Envigo, United Kingdom) were housed 3 per cage and maintained until 6 months of age at The University of Strathclyde Biological Protection Unit. All animals had ad libitum access to standard rat chow (Rat and Mouse No. 1 Maintenance, 801151, Special Diet Services, United Kingdom) and water. A 12:12 light/dark cycle was used with a temperature range of 19 to 23 °C (set point 21 °C) and humidity levels between 45% and 65%. Animals were kept in RC2F cages (North Kent Plastic, United Kingdom) with aspen wood chew sticks and hanging huts for enrichment. At 6 months of age, animals were euthanized by cervical dislocation with secondary confirmation via decapitation in accordance with Schedule 1 of the Animals (Scientific Procedures) Act 1986. Only male rats were used to limit variability, and 1 WKY rat died before reaching 6 months of age and was not included in the study.

### Experimental Techniques

In vivo blood pressure was monitored via tail cuff plethysmography (Visitech Systems BP-2000). Small mesenteric artery vascular reactivity and Ca^2+^ activity were studied using flat-mounted (*en face*) artery preparations or freshly isolated smooth muscle cells. For vascular reactivity experiments, we used the open source blood vessel diameter measurement software, Vasotracker Offline Analyzer,^32^ and recorded vascular tone in response to various pharmacological treatments. For Ca^2+^ imaging experiments, endothelial cells were preferentially loaded with the Ca^2+^ indicator, Cal 520/AM (5 µmol/L). Ca^2+^ responses were evoked by agonists or photolysis of caged IP_3_.^33,34^ Images were acquired using various epifluorescence microscopes optimized for low-light Ca^2+^ imaging using µManager microscope control software.^35^

All data presented were processed as illustrated in Figure S1 using custom Python software.^10,36–38^ Protein expression was visualized using immunofluorescence staining of mesenteric artery rings and the following antibodies: anti-alpha smooth muscle actin (cyanine 3-conjugated, catalogue No. C6198, Sigma, 1:200, raised in mouse), anti-von Willebrand factor (fluorescein isothiocyanate-conjugated, catalogue No. AB8822, Abcam, 1:50, raised in sheep), anti-CD31 (cluster of differentiation 31; platelet endothelial cell adhesion molecule, No. AF3628, R&D Systems, 1:1000 dilution, raised in goat), anti-TRPV4R (No. ACC-034, Alomone Labs, 1:1000 dilution, raised in rabbit), and anti-IP_3_R (catalogue No. 07-1210, Millipore, 1:100 dilution, raised in rabbit). The expression of TRVP4 was also quantified by immunoblotting^39^ on lysates prepared from control and hypertensive tissues using the anti-TRVP4 antibody. Endothelial cell ion channel expression was assessed using transcriptomic analysis of single-cell RNA sequencing data from mesenteric arteries generated by Cheng et al^40^ and Python-based bioinformatic tools.^41^

### Statistics and Data Analysis

Summary data are presented in text as mean±SD and graphically as individual data points mean±SD overlaid. Data were analyzed using independent 2-sample *t* tests (with Welch correction as appropriate), ordinary or repeated measures 2-way ANOVA with Sidak multiple comparisons test as appropriate, and as indicated in the respective figure or table legend. All statistical tests were 2-sided. A *P* value of <0.05 was considered statistically significant.

## Results

### Enhanced TRPV4 Channel Activity in Hypertension Is a Double-Edged Sword

**Figure 1.**
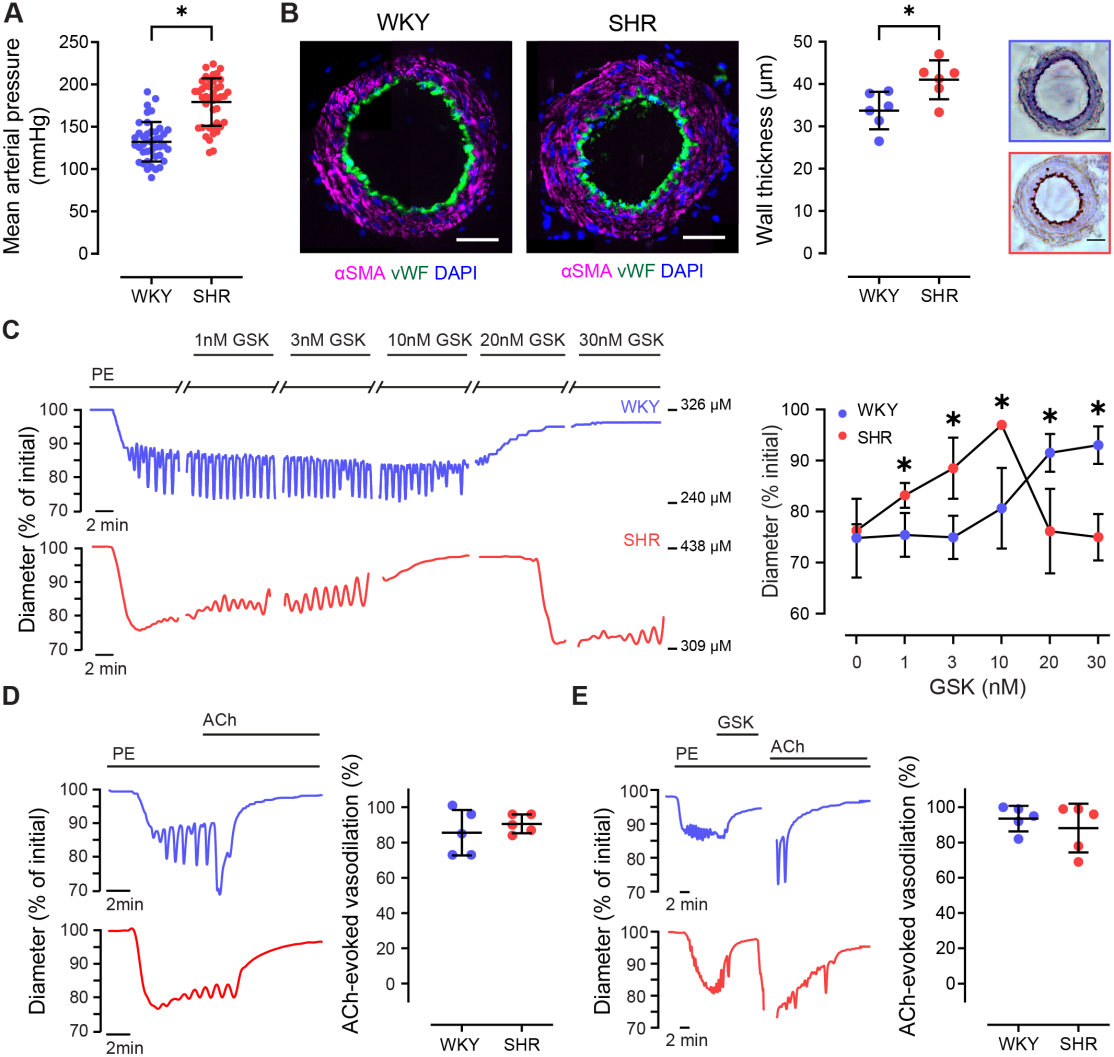
**TRPV4 (transient receptor potential vanilloid 4)-mediated vascular responsivity is altered in hypertension. A**, Mean arterial blood pressure in spontaneously hypertensive rats (SHR) and normotensive Wistar-Kyoto (WKY) control animals. **B**, Representative immunofluorescence/immunohistological images of mesenteric artery cross sections and summary data plot showing mesenteric artery wall thickness. **C** through **E**, Representative artery diameter traces and summary data (mean±SD) showing the effect of TRPV4 channel activation (using GSK1016790A [GSK], **C**) and muscarinic receptor activation (using acetylcholine [ACh], 100 nmol/L, **D** and **E**) on vascular tone in arteries preconstricted with phenylephrine (PE, concentration adjusted to achieve ~20% contraction; n=5 for each). In **D**, arteries were stimulated with ACh before any pharmacological manipulation of TRPV4 channels. In **E**, arteries were stimulated with ACh after activation of TRPV4 channels with GSK (30 nmol/L). Significance markers indicate statistical significance (*P*<0.05) using Student *t* test with Welch correction (**A**, **B**, **D**, and **E**) or 1-way ANOVA with Tukey post hoc test (**C**). Image scale bars=50 µm. DAPI indicates 4′,6-diamidino-2-phenylindole; αSMA, alpha smooth muscle acting; and vWF, von Willebrand factor.

We investigated the involvement of TRPV4 channels in the vascular changes occurring in a genetic model of hypertension (SHR). Notably, hypertensive SHR rats exhibited elevated blood pressure compared with normotensive WKY controls (Figure 1A). Similarly, arterial wall thickness was increased in the hypertensive strain compared with normotensive strain (Figure 1B).

To examine TRPV4-mediated regulation of blood vessel diameter in hypertension, we assessed vascular reactivity in small mesenteric arteries. In arteries from normotensive WKY, the specific TRPV4 channel activator GSK1016790A (GSK) evoked a concentration-dependent relaxation (Figure 1C). However, in the hypertensive strain, the response to GSK was biphasic. Low concentrations of GSK (≤10 nmol/L) induced vasorelaxation that was significantly greater than those in the normotensive strain (Figure 1C). At higher concentrations, there was a reversal of this effect, leading to constriction of the arteries. The TRPV4 channel blocker, ruthenium red, had no effect on PE-evoked vasoconstriction, but it effectively blocked the response to GSK (Figure S2), confirming the role of TRPV4 channels in the GSK-evoked vascular response. The observed increase in TRPV4-mediated relaxation and the biphasic response to TRPV4 activation in arteries from SHR animals (initial relaxation followed by a reversal to contraction at higher concentrations) suggest a complex regulatory mechanism involving TRPV4 in hypertension.

TRPV4-mediated vasodilation required an intact endothelium in WKY and SHR animals (Figure S3). Additionally, the reversal of relaxation at higher concentrations of GSK was not due to direct activation of smooth muscle, as GSK (30 nmol/L) did not evoke contraction in arteries in which the endothelium had been removed (Figure S4A and S4B). Neither did GSK evoke a Ca^2+^ increase in isolated smooth muscle cells (Figure S4C) nor in SMC from endothelium-denuded intact arteries (Figure S4D). The observed variation in TRPV4-mediated responses is specific to the TRPV4 pathway and does not reflect a general alteration in endothelial function, as endothelium-dependent relaxation evoked by acetylcholine was similar in WKY and SHR animals (Figure 1D). Furthermore, vasoconstriction to higher levels of TRPV4 activation did not arise from a nonspecific inhibition of endothelial cell function, as endothelial reactivity to acetylcholine remained intact after the occurrence of the biphasic response (Figure 1E).

These results collectively indicate that there is increased endothelial sensitivity to TRPV4 activation in hypertension, leading to greater vasorelaxation in arteries from hypertensive animals compared with normotensive controls. Furthermore, at high levels of TRPV4 activation, arteries from hypertensive animals exhibit a secondary response that is characterized by a reversal of the initial vasorelaxation response.

### Distinct Signaling Pathways

Next, we sought to elucidate the mechanisms underlying TRPV4-mediated responses in hypertension. Previous findings have indicated that TRPV4 channel activity increases endothelial Ca^2+^ levels to drive vasorelaxation.^18,42,43^ Given our observations that high levels of TRPV4-mediated activation reverse vasorelaxation, we speculated that endothelial Ca^2+^ levels might decrease at higher GSK concentrations in hypertension.

**Figure 2.**
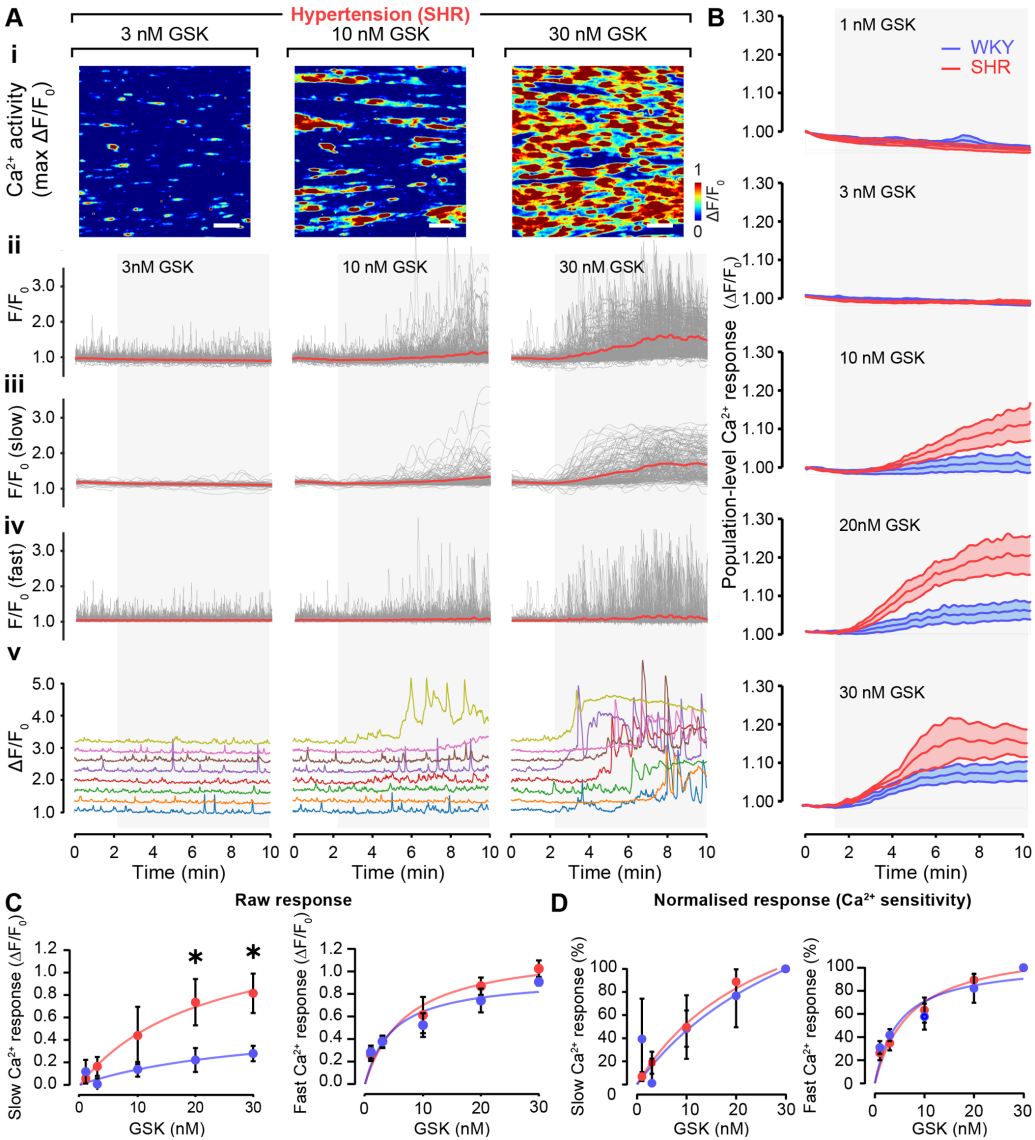
**Hypertension impairs TRPV4 (transient receptor potential vanilloid 4)-mediated endothelial Ca^2+^ signaling. Ai**, Representative images showing TRPV4-mediated Ca^2+^ activity (ΔF/F_0_ maximum intensity projections show total Ca^2+^ activity) in endothelial cells from hypertensive (SHR) animals. The Ca^2+^ responses were evoked by the TRPV4 activator GSK1016790A (GSK). Three example concentrations (3, 10, and 30 nmol/L; from the 5 used) of GSK on a single preparation are shown. **Aii** through **Av**, (**Aii**) Ca^2+^ traces (gray) from each single endothelial cell in the images in **Ai** are shown. The traces in **Aii** show the raw F/F_0_ signals, which comprised fast and slow components. The red line is the averaged signal from all cells. The isolated slow sustained component (**Aiii**), the isolated fast oscillatory component (**Aiv**), and separated individual signals from 8 randomly selected individual cells showing slow and fast signals (**Av**). **B**, Whole field-of-view average (mean±SEM; n=6) endothelial Ca^2+^ signals from normotensive (blue WKY) and hypertensive (red SHR) animals to each GSK concentration. **C**, Mean (±SD) summary data showing the amplitude of the isolated slow sustained component signals (left) and fast oscillatory signals (right) to each GSK concentration from normotensive (blue) and hypertensive (red) animals. **D**, As in **C** but normalized to the maximum response to illustrate sensitivity to the TRPV4 activator (GSK). Significance markers indicate statistical significance (*P*<0.05) using Student *t* test with Welch’s correction (n=6). Image scale bars=50 µm.

To test this hypothesis, we examined TRPV4-mediated Ca^2+^ signaling in large populations of endothelial cells using wide-field imaging (Figure 2A). In these experiments, the concentration-dependence of the endothelial responses to the TRPV4 activator GSK (1–30 nmol/L) was examined in hundreds of cells from single arteries from WKY and SHR animals. In response to each concentration of GSK, the Ca^2+^ signals from each cell were individually extracted using a largely automated image-processing procedure (Figure S1).^16,36,44,45^ The Ca^2+^ signals from each cell differed significantly in their time of occurrence, duration, and amplitude, giving rise to a substantial spread of Ca^2+^ elevations among cells to each GSK concentration (Figure 2Aii). Furthermore, within each cell, two features of the Ca^2+^ signals were evident: a slow, sustained increase in Ca^2+^ and repetitive oscillations (Figure 2Av; Video S1).

To evaluate the overall response to TRPV4 activation, we first examined average measurements obtained from all cells (Figure 2Aii) or from whole-field average measurements of endothelial Ca^2+^ (Figure 2B). These measures, which provide a consensus measure of both signaling components across the cell population, revealed that TRPV4 activation generated a larger concentration-dependent increase in endothelial cell Ca^2+^ in hypertensive animals when compared with normotensive controls (Figure 2B). This surprising finding raised the question of why do greater Ca^2+^ increases lead to a reversal of the vasorelaxation response at higher GSK concentrations in hypertensive animals but not in normotensive controls?

Given that Ca^2+^ influx is associated with endothelium-dependent vasoconstriction in hypertension,^46^ while IP_3_-mediated Ca^2+^ activity is linked to endothelium-dependent vasodilation, we speculated that differences in the contribution of Ca^2+^ influx and Ca^2+^ release within individual cells may explain our findings. Specifically, we hypothesized that the reversal of TRPV4-mediated vasodilation at high levels of TRPV4 activation occurred as a result of a shift in the dominant component of the signal moving from fast repetitive IP_3_-mediated Ca^2+^ increases to slow sustained Ca^2+^ influx (Video S1).

**Figure 3.**
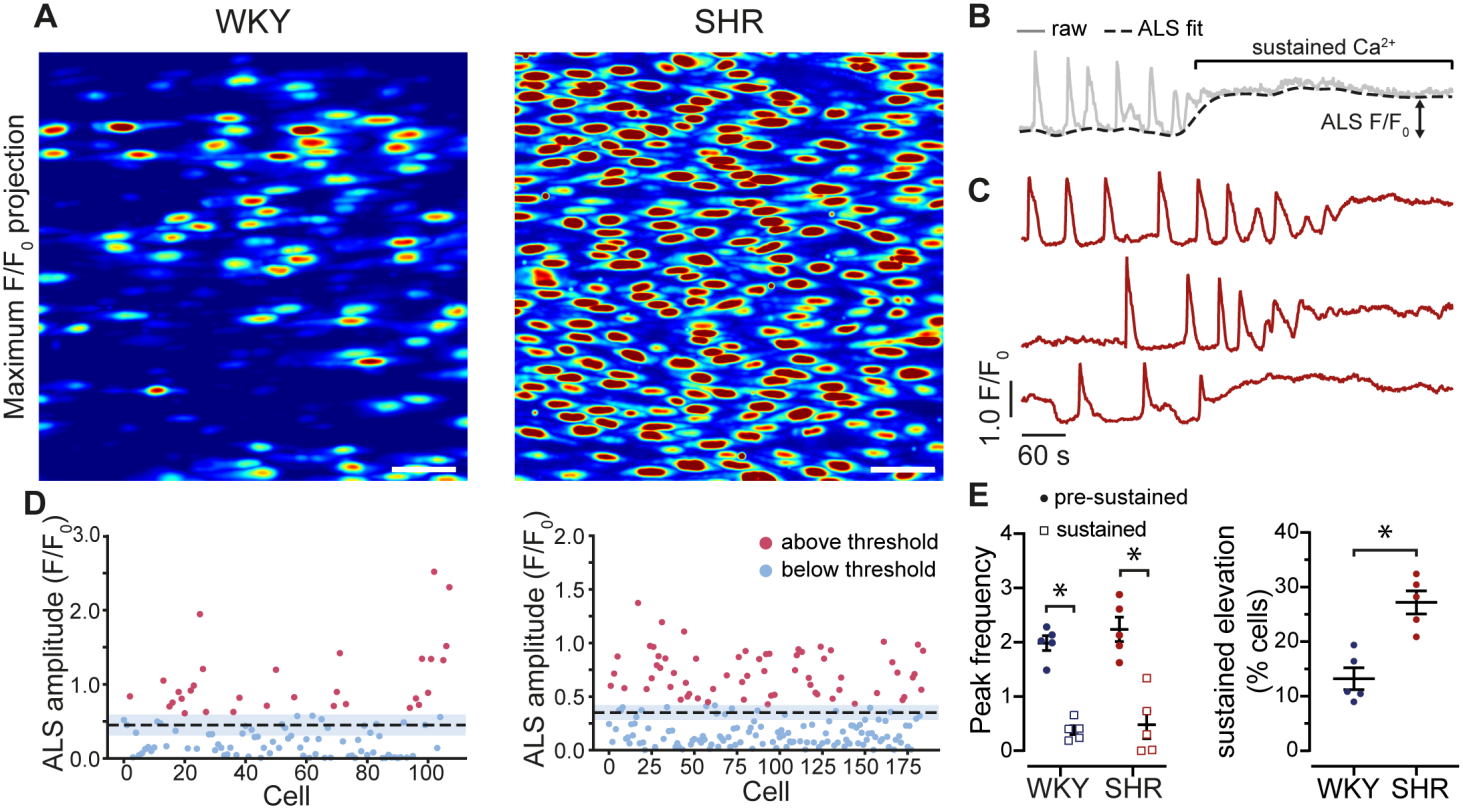
**Hypertension alters the balance between Ca^2+^ influx and Ca^2+^ release. A**, Images showing TRPV4 (transient receptor potential vanilloid 4)-mediated Ca^2+^ activity (ΔF/F_0_ maximum intensity projections, ie, total Ca^2+^ responses) in endothelial cells from normotensive (WKY) and hypertensive (SHR) animals. The Ca^2+^ responses were evoked by the TRPV4 channel activator GSK1016790A (GSK, 30 nmol/L). **B**, An example of Ca^2+^ trace (grey) from a single cell. The black dashed line is an asymmetric least squares (ALS) fit to the Ca^2+^ signal, showing that the ALS fit tracks the slow, sustained Ca^2+^ change. **C**, Three Ca^2+^ traces from hypertensive animals (SHR) illustrating the shift from oscillatory to sustained elevated Ca^2+^ levels after TRPV4 activation. **D**, The average amplitude of the Ca^2+^ signal from all cells in **A** from normotensive (WKY, **left**) and hypertensive (SHR, **right**) animals. The last 30 seconds of the ALS fit determine the average amplitude. The mean amplitude, of all cells, is represented by a black dashed line, and the SE is shown as the blue shaded area. Signals above the threshold (see methods) are shown in red and those below the threshold in blue. **E**, **Left**, Summary data illustrating the switch from a transient oscillatory (presustained) to the slow sustained Ca^2+^ response to TRPV4 channel activation. **Right**, There is an increased percentage of cells that exhibit the switch to a slow sustained Ca^2+^ signal in hypertension when compared with normotensive controls. Data are mean±SD (n=5 per group). Significance markers indicate statistical significance (*P*<0.05) using Student *t* test with Welch’s correction. Image scale bars=50 µm.

To investigate this hypothesis, we examined the influx and release components of each cell’s Ca^2+^ signal to determine each of their contributions to the overall response. The slow, persistent elevation is consistent with Ca^2+^ influx, while rapid oscillations reflect Ca^2+^ release from the internal store.^18,43,47^ The Ca^2+^ increases in each cell were therefore separated based on the kinetics of the signals (Figure 2A; Figure S1). Each signal’s slow, persistent elevation was isolated from the overall response by using an asymmetric least squares fit to the data. The asymmetric least squares fit tracks the slow, persistent change in Ca^2+^ and is unaffected by rapid oscillations (Figure 3B). The fast oscillatory component was separated from the overall Ca^2+^ change by normalizing each signal with its asymmetric least square-smoothed counterpart (see Methods section; Figure S1). This approach completely separates slow sustained Ca^2+^ changes and rapid oscillatory Ca^2+^ increases for each cell for subsequent analysis (Figure 2Aii through 2Aiv).

The amplitude of each component increased with the level of TRPV4 activation. Consistent with our hypothesis, the amplitude of the response was significantly higher in hypertension than in normotensive controls, while there was no difference in the amplitude of the fast component (Figure 2C). This alteration was not due to changes in sensitivity to the activator, GSK, as there was no difference in either component when normalized to the maximum response (Figure 2D). Once again, the results do not reflect a generalized alteration in endothelial function since endothelial Ca^2+^ responses evoked by 3 distinct mechanisms—muscarinic receptor activation, emptying of internal Ca^2+^ stores, and direct activation of IP_3_ receptors—were each similar in the 2 rat strains (Figures S5 and S6).

On the basis of these observations, we next performed additional analysis on the Ca^2+^ response evoked in each cell upon TRPV4 activation (Figure 3). Our analysis revealed a distinct shift in the distribution of Ca^2+^ signaling modes in individual cells to that of predominately slow sustained responses in SHR.

In both WKY and SHR, the initial response to TRPV4 activation was characterized by Ca^2+^ oscillations (Figure 3A and 3B). However, a subset of cells transitioned from this oscillatory pattern to a sustained Ca^2+^ increase with a high plateau (Figure 3C). As cells switched to a sustained response, the mean frequency of oscillations reduced, and the majority of cells exhibited minimal discernible oscillations (Figure 3C). These data are consistent with a transition from IP_3_-mediated Ca^2+^ release to Ca^2+^ influx. Of significant interest, the number of cells that underwent the transition from oscillatory responses to sustained Ca^2+^ influx was increased in arteries from hypertensive animals (Figure 3D and 3E). In this analysis, we examined the mean asymmetric least squares response across all cells (Figure 3D, black dotted line). A threshold value was defined as 3× the SEM amplitude for the field of endothelial cells in each experiment. Significantly more endothelial cells from SHR than WKY exceed this threshold value (Figure 3D, red dots; Figure 3E, right panel). These results suggest that while the switch from release to influx occurs in both WKY and SHR, there is an increased number of cells showing sustained Ca^2+^ influx in hypertension (Video S1).

Collectively, our findings reveal that vasodilator responses may arise largely from IP_3_-mediated Ca^2+^ activity and that the shift to vasoconstriction in hypertension may be attributed to a transition to a sustained Ca^2+^ influx in endothelial cells.

### TRPV4 Channel Expression Is Upregulated in Hypertension

**Figure 4.**
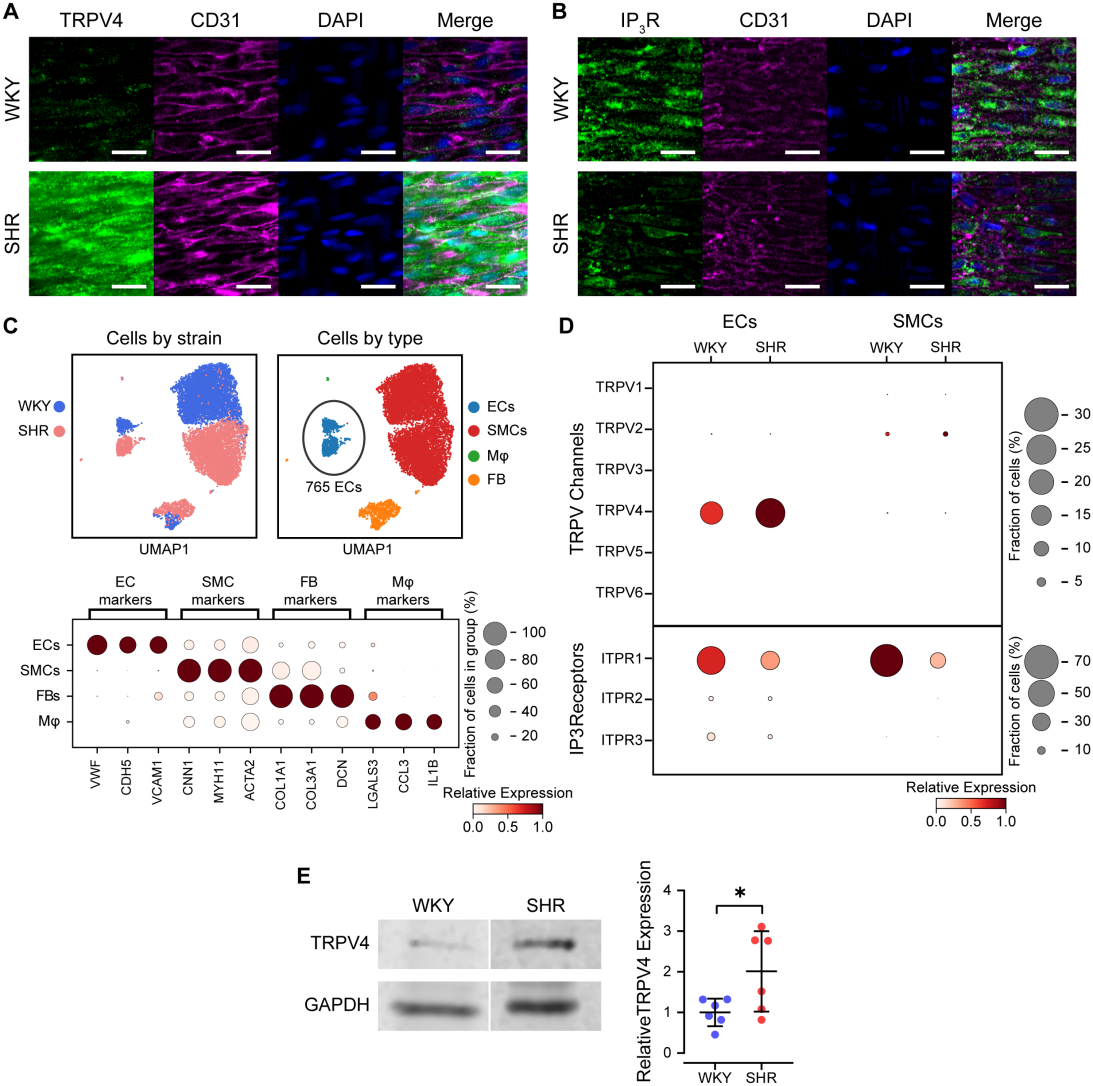
**Endothelial TRPV4 (transient receptor potential vanilloid 4) channel and IP_3_ (inositol triphosphate) receptor expression are altered in hypertension. A** and **B**, Endothelial cell ion channel expression in normotensive (WKY) and hypertensive (SHR) rats revealed by in situ fluorescence imaging. Images show TRPV4 (**A**) or IP_3_ (**B**) receptor (green), cell-cell borders (CD31 [cluster of differentiation 31], magenta), and nuclei (4′,6-diamidino-2-phenylindole [DAPI], blue). All staining was performed simultaneously in n=3 samples from both WKY and SHR, using pooled antibody that was aliquoted between the samples. Imaging was performed simultaneously, using the same microscope settings, and all images were processed identically. All images shown are contrast-matched. No fluorescence signal was detected in arteries incubated with secondary antibodies in the absence of any primary antibody (Figure S7). Scale bars=25 µm. **C** and **D**, Single-cell transcriptomic analysis of vascular cells from mesenteric arteries of normotensive and hypertensive rats. In total, 12 161 expression profiles were analyzed. **C**, Uniform manifold approximation (UMAP) projection embeddings (**top**) and dot plot analysis (**bottom**) of cell-specific marker genes. **D**, Dotplot of TRPV and IP_3_ receptor isoform expression in endothelial cells and smooth muscle cells. In UMAP plots, data from individual cells are colored by animal strain (WKY, blue; SHR, pink) or annotated cell type (endothelial cell [EC], smooth muscle cell [SMC], fibroblasts [FB], and macrophages [Mφ]). In dot plots, the size of the dot indicates the percentage of cells in a group in which the indicated gene was detected, and the color represents the relative expression level of that gene in the indicated group. Single-cell sequencing data obtained from Gene Expression Omnibus (GSE149777); see Tables S1 and S2 for differential expression analysis.^40^ **E**, **Left**, Western blot analysis of TRPV4 protein expression in mesenteric arteries from WKY and SHR animals. **Right**, Mean±SD of TRPV4 expression in WKY and SHR animal mesenteric arteries normalized to GAPDH.

We next investigated the possibility that the hypertension-induced alterations in TRPV4 function and signaling were paralleled by changes in the endothelial cell transcriptome. Specifically, we hypothesized that the expression of TRPV4 ion channels would be increased in endothelial cells of hypertensive animals. Immunostaining of endothelial cells (confirmed by the cell adhesion label CD31) revealed a diffuse expression pattern of both TRPV4 ion channels and IP_3_ receptors (Figure 4A and 4B). This expression pattern was not observed when the secondary antibody was omitted (Figure S7). In line with our initial hypothesis, the endothelial TRPV4 fluorescence signal appeared to be higher in hypertensive animals when compared with the normotensive control group (Figure 4A). However, IP_3_ receptor expression was reduced (Figure 4B).

To further test this hypothesis, we analyzed single-cell RNA data from mesenteric arteries generated by Cheng et al.^40^ The data set contains the expression levels of over 20 000 genes from over 12 000 mesenteric artery cells isolated from hypertensive, SHR, and normotensive, WKY rats. Using these data, we performed automated cell-type annotation and extracted transcriptomic data from 765 high-quality endothelial cells (Figure 4C and 4D). These cells have robust expression of the canonical endothelial cell-specific marker genes von Willebrand factor, cadherin 5, and vascular cell adhesion molecule 1, with minimal expression of smooth muscle, fibroblast, or macrophage markers (Figure 4C). TRPV4 and IP_3_ receptor expression correlated with our immunostaining results (Figure 4D)—endothelial TRPV4 expression was ≈60% higher in hypertensive versus normotensive control rats, while IP_3_ receptor expression was ≈60% lower in the hypertensive strain. Additionally, TRPV4 channel expression was minimal in smooth muscle cells (see also 24,40,48–50). TRPV4 protein levels were found to be higher in SHR mesenteric arteries compared with those from WKY, as confirmed by Western blot analysis, consistent with the findings from single-cell sequencing and immunostaining (Figure 4E; Figure S8).

Together, these results suggest that the augmented vascular responses observed in hypertension arise from upregulated expression of plasmalemmal TRPV4 channels. The decreased expression of IP_3_ receptors may explain the reduction in endothelial control of basal vascular tone previously reported to occur in hypertensive rats.^51^

## Discussion

Here, we show there is increased TRPV4 channel expression and activity in hypertensive animals when compared with normotensive controls. Since there is no change in TRPV4 sensitivity to pharmacological activation, increased expression of the channel appears sufficient in explaining the heightened Ca^2+^ signaling on channel activation. The consequences of increased TRPV4-mediated Ca^2+^ signaling in hypertension are complex. At low levels of channel activation, there is increased vasodilation when compared with normotensive controls. This observation is consistent with the increased expression of the channel and Ca^2+^ signaling that occurs in hypertension. However, paradoxically, the increased Ca^2+^ signaling that occurs at higher levels of channel activation resulted in a reversal of endothelium-dependent dilation and vascular contraction. These results suggest that altered TRPV4 activity in hypertension may either be protective at low levels of channel activity or a contributor to the progression of hypertension at high levels of channel activity.

The question arises as to why different types of contractile responses occur to the Ca^2+^ signaling events evoked by TRPV4 activation in hypertension. An explanation is found in the distinctive nature of the Ca^2+^ signals generated at low and high levels of TRPV4 activity. The signals generated by TRPV4 activation consist of 2 major components: a slow, sustained response and rapid transient Ca^2+^ oscillations. The slow component arises from Ca^2+^ influx via TRPV4, which generates the sustained Ca^2+^ increase. At low levels of activation, this sustained rise is relatively small. The sustained rise is amplified by Ca^2+^-induced Ca^2+^ release acting at IP_3_ receptors. It is the latter that generates the rapid transient Ca^2+^ oscillations.^18,47^ These oscillations occur at all levels of TRPV4 activation in normotensive controls. In hypertension, the oscillations have prominence at lower levels of activation, whereas the sustained slow component dominates at higher levels of TRPV4 activity. It is tempting to speculate, therefore, that dilation is associated with rapid Ca^2+^ oscillations while contraction is promoted by the increased sustained response. Thus, there appears to be a redirection of Ca^2+^ signals to generate a different functional outcome (contraction) at higher levels of channel activity in hypertension.

The altered priority of the slow and rapid types of Ca^2+^ signal in hypertension can itself be explained by changes in the expression of TRPV4 and IP_3_ receptors. In hypertension, there is increased expression of TRPV4 and, significantly, a decreased expression of IP_3_ receptors. It is IP_3_ receptor activity that underlies the Ca^2+^ oscillations and dilation. These results suggest that, in hypertension, as the extent of TRPV4 activation increased, so did the slow sustained component. At the same time as the sustained component increases, the relative contribution from rapid oscillations associated with IP_3_ receptors decreases because of the reduced expression of the receptor.

In hypertension, at lower levels of TRPV4 activity, there is increased dilation when compared with normotensive controls. The IP_3_ response, which underlies the relaxation, is increased. Yet IP_3_ receptor expression is reduced, and the store content is unaltered in hypertension. The event that triggers IP_3_-evoked Ca^2+^ release is Ca^2+^ influx via TRPV4. TRPV4 expression and distribution are substantially increased in hypertension. It seems likely that the increased event triggering IP_3_-evoked Ca^2+^ release (TRPV4-mediated Ca^2+^ influx) is sufficient to offset the decreased expression of IP_3_ receptors—at least at lower levels of activation. Alternatively, it is possible that many IP_3_ receptors are nonfunctional when it comes to triggering Ca^2+^ release in response to IP_3_.^52^

Since TRPV4 channels are a major route for Ca^2+^ entry in endothelial cells, they are widely regarded as promoters of nitric oxide production and in activating small- and intermediate-conductance Ca^2+^-sensitive potassium channel activity. Generation of nitric oxide and activation of small- and intermediate-conductance Ca^2+^-sensitive potassium results in vasodilation and decreased vascular resistance. In hypertension, there is chronically decreased nitric oxide production and reduced small- and intermediate-conductance Ca^2+^-sensitive potassium channel activity, which leads to impaired endothelial-dependent dilation. These observations resulted in the hypothesis that decreased Ca^2+^ entry via TRPV4 may be a component of the changes associated with hypertension. Yet the relationship between Ca^2+^ entry via TRPV4 channels and the vascular changes that accompany hypertension is disputed. There are reports of changes in the channel’s activity that result in increased vasodilation, decreased vasodilation, increased contraction, or no contribution at all. For example, in hypertension, TRPV4 expression may be reduced and, as a result, channel activity impaired. The decreased channel activity is proposed to contribute to the increased vascular resistance that leads to elevated blood pressure since channel activity was associated with vasodilation.^23–25^ However, in animals in which the channel has been knocked out, blood pressure may, paradoxically, be lower than controls.^28^ In other studies, rather than being decreased, TRPV4 expression and channel activity may be increased in hypertension. The increased TRPV4 activity promotes vasodilation and is proposed to provide protection against the vascular changes that occur in hypertension.^26^ Alternatively, rather than underlying dilation, increased TRPV4 expression and Ca^2+^ influx may promote endothelium-dependent contraction in hypertension. The increased activity of the channel and its associated contraction is proposed to be a contributory factor that leads to the increased vascular resistance that characterizes hypertension.^27^ In yet other studies, TRPV4 channel knockout mice do not show increased blood pressure under resting conditions, as may be expected if the channel is significant in regulating vascular resistance.^29,30^ While many studies show a parallel association between altered expression of TRPV4 and functional outcomes, some studies report unaltered expression (as measured by current density) but diminished functional outcome as a result of decoupling of the TRPV4 from an effector (the KCa2.3 [small conductance calcium-activated potassium channel 3] channel) in hypertension.^53^

Thus, there is an uncertain relationship between TRVP4 activity and the vascular changes that occur in hypertension. Our study offers at least a partial explanation for the apparently contradictory findings by highlighting various ways in which TRPV4 may alter arterial activity in precisely the same artery. We show that oscillatory Ca^2+^ signals associated with IP_3_-evoked Ca^2+^ release, triggered by TRPV4 activation, generate relaxation. The sustained Ca^2+^ signals occurring at high levels of TRPV4 activation in hypertension appear to elicit contraction as a result of reduced endothelium-dependent relaxation. It is the increased expression of TRPV4 and decreased expression of IP_3_ receptors that predisposes the artery to sustained Ca^2+^ rises and contractions that occur at high levels of TRPV4 activation in hypertension.

The question arises as to how TRPV4-mediated Ca^2+^ entry into the endothelial cytoplasm can trigger arterial relaxation in some conditions and decreased relaxation and contraction in others. Ca^2+^ has the potential to activate numerous, and at times, conflicting, processes in cells. Ca^2+^-dependent functional activities are normally coordinated to avoid conflict via timely and spatially variant Ca^2+^ signals that are matched to control observable responses. For example, the extent of activation of effectors may rely on highly local Ca^2+^ elevations or on the duration, amplitude, or repetitiveness of transient global or local increases in the concentration of the ion. The specific outcomes resulting from increased Ca^2+^, such as effects on calmodulin, nitric oxide synthase, phospholipase A2 activation, and calmodulin-dependent kinase II, depend on the rates of ion binding to the effector (including on and off rates). The temporal aspects of Ca^2+^ concentration change, coupled with effector on and off rates, play a crucial role in determining whether or not the timeframe permits accumulated activity to encode both frequency and amplitude, as shown for nuclear factor of activated T cells translocation.^54^ The large number of Ca^2+^-binding proteins with unique on and off rates for Ca^2+^ binding is critical in determining functional outcomes, as appears to be the case in the present study.

Ca^2+^-activated increases in nitric oxide production and small- and intermediate-conductance Ca^2+^-sensitive potassium channel activity trigger relaxation. An endothelium-dependent contraction, or decreased endothelium-dependent relaxation, may be evoked by several Ca^2+^-dependent mechanisms. Sustained Ca^2+^ rises, for example, may result in significant mitochondrial Ca^2+^ uptake. Excessive mitochondrial Ca^2+^ uptake leads to an increase in reactive oxygen species production and oxidative stress. Reactive oxygen species and oxidative stress may limit relaxation by decreasing the concentration of nitric oxide through the formation of the peroxynitrite anion. The production of additional endothelium-derived contracting factors, such as prostanoids, and endothelin is also Ca^2+^ dependent. Rises in Ca^2+^ increase expression of preproendothelin-1 mRNA via a Ca^2+^/calmodulin kinase pathway^55,56^ or prostanoids via Ca^2+^-dependent phospholipase A_2_ activation.^57^

In the present study, it seems likely that TRPV4 suppresses relaxation at higher levels of activation in hypertension (rather than cause contraction) because TRPV4 activation did not cause contraction by itself in an artery that was not preconstricted (Figure S4). Furthermore, all vascular effects of TRPV4 in the present study are mediated via the endothelium since channel activation evoked no change in arterial tone (either contraction or relaxation) in the absence of the endothelium.

## Perspectives

The proposed importance of TRPV4 to endothelial Ca^2+^ entry and changes in vascular disease have resulted in the channel being linked to the vascular changes in hypertension. However, in various studies, the changes in TRPV4 activity have been reported as being both protective and a contributor to the pathological changes that occur in hypertension. Our results help reconcile the contradictory proposals by highlighting two responses to TRPV4 activation in hypertension. At low levels of TRPV4 activity, there is increased relaxation to the activation of the channel in vessels from hypertensive animals when compared with normotensive controls. However, at higher levels of TRPV4 activation, endothelium-dependent dilation was impaired, resulting in increased contraction. These results show that at low levels of activity, TRPV4 activity may offer some protection to the vascular changes in hypertension, while at higher levels of activity, TRPV4 may contribute to the increased vascular tone that accompanies hypertension. High levels of TRPV4 activity may occur in hypertension as a result of the increased mechanical stimuli generated by increased pressure and flow velocity in small vessels, which may contribute to the vascular changes underlying hypertension.

## Article Information

### Acknowledgments

The support of the British Heart Foundation is gratefully acknowledged.

### Sources of Funding

This study was funded by the British Heart Foundation (RG/F/20/110007; PG/20/9/34859).

### Disclosures

None.

### Supplemental Material

Expanded Materials and Methods

Figures S1–S8

Video S1

References 18,32–35,39–41,43,45,51,58

## Supplementary Material


