# Supplementary material for: Increased TRPV4 Channel Expression Enhances and Impairs Blood Vessel Function in Hypertension
Source: Hypertension. 2024 Oct 23;82(1):57–68. doi: 10.1161/HYPERTENSIONAHA.124.23092 (PMC11654454; doi:10.1161/HYPERTENSIONAHA.124.23092)
Supplement: Supplementary file 2 [file hyp-82-057-s004.docx]

Supplementary Materials

Increased TRPV4 channel expression enhances and impairs blood vessel function in hypertension

Xun Zhang, Charlotte Buckley, Matthew D Lee, Christine Salaun, Margaret MacDonald, Calum Wilson, John G McCarron

Strathclyde Institute of Pharmacy and Biomedical Sciences, University of Strathclyde, 161 Cathedral Street, Glasgow G4 0RE, UK.

Keywords: hypertension, endothelial cell, TRPV4, vascular reactivity, IP_3_ receptor

**Detailed Materials and Methods**

**Blood Pressure Measurement**

Blood pressure was monitored using tail cuff plesthysmygrophy (Visitech Systems BP-2000). In each measurement session, ten measurements of heart rate, and systolic, diastolic and mean blood pressures were taken and the average of each determined. A total of 4 blood pressure determinations were made. In the initial measurements a minimum of 48hrs was left between recordings. Two further sets of blood pressure measurements were taken subsequently, one of which was the week before euthanasia. Animals were euthanized at 6 months old (~350g) by cervical dislocation.

**Chemicals**

The physiological saline solution (PSS) used in all functional experiments consisted of:- 145 mM NaCl, 2 mM MOPS, 4.7 mM KCl, 1.2 mM NaH2PO4, 5 mM Glucose, 0.02 mM EDTA, 1.17 mM MgCl, 2 mM CaCl, pH 7.4. All reagents included in the PSS were obtained from Sigma.

Cal-520/AM was obtained from Abcam (UK). Caged-IP_3_ (caged-IP_3_ 4,5-dimethoxy-2-nitrobenzyl) and Pluronic F-127 was obtained from Sichem (Germany). GSK1016790A, acetylcholine, ionomycin, cyclopiazonic acid, phenylephrine, sodium nitroprusside, ruthenium red, caffeine, and dimethyl sulfoxide (DMSO) were obtained from Sigma Aldrich (USA). All solutions were prepared fresh each day and chemicals were diluted to the desired concentration with PSS.

## En face artery preparation

Either second or third order mesenteric arteries were used for measurement of vascular reactivity or endothelial cell Ca^2+^ signaling. Immediately following euthanasia, the mesentery bed was removed and placed in PSS. Arteries were dissected rapidly, cleaned of connective tissue and fat and used immediately. Arteries were pinned to either (1) the Sylgard-coated base of a custom chamber for use on an upright microscope or (2) a Sylgard block that was subsequently placed in a custom chamber for use on an inverted microscope. The arteries were then cut open longitudinally and pinned flat with the endothelial layer facing upwards. Endothelial cells were preferentially loaded with the fluorescent Ca^2+^ indicator Cal-520/AM (5 µM with 0.04% Pluronic F127 and 0.26% dimethyl sulfoxide [DMSO] in PSS) at 37°C for 30 minutes. In a subset of experiments, endothelial cells were also loaded with a membrane-permeant, photolabile form of IP_3_. In these experiments, cIP_3_ was included in the Ca^2+^-indicator solution. Following a 30-minute incubation period, arteries were gently washed PSS and then positioned on a microscope for imaging.

**Smooth Muscle Cell Isolation**

First to fourth order mesenteric artery segments were enzymatically digested to obtain freshly isolated smooth muscle cells. In brief, vessels cut open and then into small strips of approximately 2 mm length, and then enzymatically digested using collagenase (Type 2, 256 units/mg, 2 mg.ml^-1^) in a water bath at 37^o^C for 45-60 mins. The supernatant was then removed gently and the arteries triturated using a wide-bored, fire-polished glass pipette. Cells were transferred to a glass-bottomed chamber for Ca^2+^ imaging, stained with Cal-520/AM (5 µM, 30 mins, 37^o^C) for Ca^2+^ imaging experiments.

**Immunocytochemistry**

Arteries and mounted into Sylgard-lined 6-well plates as *en face* arterial preparations. The arteries were fixed in 4% paraformaldehyde (PFA; Agar Scientific, UK) in phosphate buffered saline (PBS) for 20 mins at room temperature. Preparations were then washed three times in glycine solution (0.1 M), three times in PBS and then permeabilized with Triton-X100 (0.2% in PBS) for 30 minutes. Cells were again washed three times in PBS, three times in antibody wash solution (150 mM NaCl, 15 mM Na_3_C_6_H_5_O_7_, 0.05% Triton‐X100 in milliQ water), and incubated for one hour with blocking solution (5% donkey serum in antibody wash solution) at room temperature. All individual wash steps were 5 minutes in duration. Preparations were then incubated overnight at 4^o^C with combinations of anti-alpha smooth muscle actin (Cy3-conjugated, catalogue # C6198, Sigma, 1:200 , raised in mouse), anti-von Willebrands Factor (FITC-conjugated, Catalogue # AB8822, Abcam, 1:50, raised in sheep), goat anti-CD31 (CD31/PECAM; R&D Systems cat. #AF3628, 1:1000, raised in goat) and anti-TRPV4R (Alomone Labs, Cat. # ACC-034, 1:1000, raised in rabbit) or anti-IP_3_R (Millipore, Cat. # 07-1210, 1:100, raised in rabbit) primary antibodies diluted in antibody buffer (1:1000 dilution; 0.15 M NaCl, 15 mM Na_3_C_6_H_5_O_7_, 2% donkey serum, 1% BSA, 0.05% Triton X‐100 in milliQ water). Following primary antibody incubation, preparations were washed three times in antibody wash solution and, where required, incubated for one hour at room temperature with fluorescent secondary antibodies conjugated to Alexa Fluor 488 (donkey anti‐goat, 1:1000; A-11055 labelling anti-CD31) and Alexa Fluor 555 (donkey anti‐rabbit, 1:1000; A-31572 labelling anti-TRPV4) in antibody buffer. The preparations were then washed three times in antibody wash solution, incubated with the nuclear stain, 4′,6-diamidino-2-phenylindole (DAPI; 4 nM) for 5 mins, and finally washed three more times in PBS (5 mins) prior to imaging. All samples were processed in a single immunostaining run and were imaged using the same microscope settings.

***Detection and quantification of TRPV4 expression by immunoblotting***

Tissues from control and hypertensive rats were lysed in RIPA buffer (ThermoFisher Scientific, US) containing protease and phosphate inhibitors (ThermoFisher Scientific, US). Protein concentrations were quantified by DC protein assay (Bio-Rad, US). In brief, protein lysis and 6 dilutions of a protein standard containing 0.2 mg/ml to 6.4 mg/ml bovine serum albumin were added to a 96-well microplate. Working reagents A and B were added to each well and gently mixed on an orbital shaker for 15 min and absorbance at 750nM was read by a plate reader (HideX, Finland). Protein concentrations were calculated against the standard curve. An equal amount of total protein (30 µg) from each sample was used

and 4x Laemmli sample buffer (Bio-Rad, US) was added to the samples. Protein samples were incubated at 95 °C for 10 min, and quantification of TRPV4 expression was achieved by immunoblotting[^1^](#_ENREF_1).

The rabbit antibody against TRPV4 was from ThermoFisher Scientific (PA5-41066) and diluted at 1:500 (1 µg/ml) whereas the mouse anti GAPDH antibody was from Proteintech (#60004-1-lg) and diluted at 1:10,000 (0.1 µg/ml). Donkey anti-mouse (IR680RD; Li-Cor #926-68072) and anti-rabbit (IR800CW, Li-Cor #926-32213) antibodies were diluted at 1:20,000. Immunoblots were scanned with an Odyssey Li-Cor infrared scanner and quantified with the ImageStudio software (Li-Cor, US). The level of expression of TRPV4 was normalized to that of GAPDH.

**Assessment of vascular reactivity**

Vascular reactivity was assessed in isolated mesenteric arteries mounted *en face*[^2^](#_ENREF_2)^,^[^3^](#_ENREF_3). Arteries were visualized at 5 Hz using an upright fluorescence microscope (FN-1; Nikon, Japan) equipped with a 16X, 0.8 numerical aperture objective, 460 nm LED illumination (CoolLED, UK), and an iXon 888 (Andor, UK) electron multiplying CCD camera. The resulting 832 x 832 µm field of view allowed quantification of vascular reactivity in opened arteries using VasoTracker edge-detection algorithms[^4^](#_ENREF_4). Contraction data are represented as the percent reduction from resting diameter. Relaxation data (from constricted diameter) are represented as the percent of maximal relaxation (constricted diameter to resting diameter).

Arteries were partially constricted with phenylephrine added to the perfusate (to ~80% of resting diameter i.e. 20% contraction; ~2 µM phenylephrine). This level of constriction enables the vessels to either dilate or constrict further under experimental pharmacological studies[^5^](#_ENREF_5). Arteries were then assessed for endothelium-dependent relaxation to the muscarinic receptor agonist, acetylcholine (100 nM). All arteries exhibited a relaxation greater than 70% of the maximum possible, were considered viable and used in subsequent experiments. Following washout, arteries were preconstricted once more and then responses to increasing concentrations of the TRPV4 channel agonist, GSK1016790A (GSK), were examined. In a subset of experiments, the effect of endothelium removal was assessed. In these experiments, the endothelium was removed by gently scraping the intimal surface with a fine hair and smooth muscle cell viability was confirmed using phenylephrine and sodium nitroprusside (100 µM). In an additional series of experiments, the effect of the TRPV4-channel blocker, ruthenium red (5 µM), on GSK-evoked vascular responses was assessed. In these experiments, arteries were first preconstricted with phenylephrine. Ruthenium red was then added to the perfusate for 10 minutes prior to the addition of GSK1016709A. All arteries used for experimentation had a luminal diameter ~150 µm, and were perfused with PSS (37^o^C) at a rate of 1.5 ml min^-1^ using a Gilson Minipuls 3 peristaltic pump.

## Endothelial cell Ca^2+^ imaging

Intact mesenteric artery endothelial cell Ca^2+^ activity was recorded at 10 Hz using one of three imaging systems optimized for high-resolution Ca^2+^ imaging: an upright epifluorescence microscope (described above; FN-1, Nikon, Japan) equipped with a 16X 0.8 NA objective lens, and two inverted fluorescence microscopes (TE300 or Ti-Eclipse, Nikon, Japan) each equipped with 40X and 100X oil-immersion (1.3 NA; S Fluor) objective. The TE300 microscope was also equipped with a flash lamp for localized spot photolysis (00-325-JML-C2; Rapp Optoelectronics, Germany). All microscopes were fitted with multi-band LED illumination systems (pE-300 or pE-4000; CoolLED, UK), multi-band filter cubes (UV/FITC/TRITC), and sensitive, large-format EMCCD camera (iXon Ultras, 1024 by 1024 pixels; Andor, UK). All images were acquired using µManager microscope control software[^6^](#_ENREF_6).

**Ca^2+^ imaging protocols**

In all experiments, responses to the muscarinic receptor agonist, acetylcholine (100 nM, perfused at 1.5 ml min^-1^), were first assessed to confirm endothelial viability and physiological function. Following washout of acetylcholine, and a 10-minute re-equilibration period, endothelial cell Ca^2+^ response to increasing concentrations of GSK were then examined. In experiments assessing Ca^2+^ store content, arteries were perfused with Ca^2+^ free PSS for 5 min to remove external Ca^2+^. Cyclopiazonic acid (20 µM) was then added to the Ca^2+^-free perfusate. In experiments examining Ca^2+^ responses evoked by direct activation of IP_3_ receptors, the inositide was released (caged IP_3_) using a UV flash of ~ 1 ms duration[^7^](#_ENREF_7)^,^[^8^](#_ENREF_8). A broadband light source coupled to the to the epi-illuminator allowed the position of the uncaging region (~ 70 µm diameter) and which endothelial cells were directly activated by the spot photolysis system to be determined. Three recordings of responses to uncaging of IP_3_ were recorded from each biological replicate, with a 15 mins recovery period between flashes to allow Ca^2+^ store refilling to occur.

In all Ca^2+^ imaging experiments, endothelial cells were activated after a minimum baseline recording of 1 minute in duration (pharmacological activation) or 30 seconds (spot photolysis). All images were acquired at 10 Hz.

**Analysis of Ca^2+^ activity**

Single-cell endothelial Ca^2+^ activity was assessed as previously described[^5^](#_ENREF_5)^,^[^9^](#_ENREF_9)^,^[^10^](#_ENREF_10) and outlined in Fig S1. In brief, we used automated algorithms to extract fluorescence intensity as a function of time from circular regions of interest (~15 µm diameter) centered on each cell in our images. Fluorescence signals were then smoothed using a Savitzsky-Golay (21 point, third-order) filter, and expressed as fractional changes in fluorescence (F/F_0_) from baseline (F_0_). The baseline was automatically determined by averaging the fluorescence intensity of the 100-frame portion of each trace that exhibited the least noise. We then calculated the discrete derivative (d(F/F_0_)/dt) of each Ca^2+^ signal, and used a peak-detection algorithm to identify increases in fluorescence intensity that rose at least 15 SD above baseline noise. For acetylcholine-evoked responses, Ca^2+^ activity was quantified using the number (or percentage) of cells exhibiting spiking activity, and the amplitude of these Ca^2+^ spikes (ΔF/F_0_). Analysis of IP_3_-evoked Ca^2+^ activity was restricted to those cells in which cIP_3_ was released by applying a mask restricted to the photolysis region**.**

The response to TRPV4 channel activation involves two main components: (1) a slow, sustained elevation in baseline Ca^2+^ levels and (2) rapid intracellular Ca^2+^ waves (oscillations). To separate these components, each signal's slow persistent elevation was isolated using an asymmetric least squares (ALS) smoothing technique. The fast-oscillatory signal component was separated by normalizing each signal with its ALS-smoothed counterpart to eliminate slow drifts, and signals processed as above. To give an indication of the percentage of cells exhibiting sustained elevations in Ca^2+^ levels, we calculated the start of a sustained elevation in Ca^2+^ as 90% of the ALS slope. The mean amplitude and frequency of events were measured from this point until the end of the recording. The mean amplitude (+3x standard error of the mean) was used as a threshold to define cells with the highest sustained response.

To assess the internal store content (experiments using cycolopiazonic acid), entire field-of-view average Ca^2+^ signals were extracted using ImageJ and plotted in Origin Pro (OriginLab Corporation, US). In brief, Ca^2+^ traces were plotted and the area under the curve (AUC) calculated as a measure of total internal store content.

For graphical representations of endothelial cell Ca^2+^ activity, we created single image representations of Ca^2+^ recordings. This image was created by taking the maximum intensity of F/F_0_ image sequences for the duration of the recordings. In the case of caged IP_3_-evoked Ca^2+^ experiments, the image was created from the first 2 s (20 frames) immediately following IP_3_ uncaging and presented using a JET LUT.

**Single cell RNA Sequencing Data Analysis**

We obtained Smart-Seq2 RNA sequencing libraries of single mesenteric artery cells submitted by Cheng *et al.* from the Gene Expression Omnibus database ([GSE149777](https://www.ncbi.nlm.nih.gov/geo/query/acc.cgi?acc=GSE149777))[^11^](#_ENREF_11). The dataset contains the read count matrix of 25340 genes from cells isolated from arteries from WKY (7197 cells) and SHR (6549 cells) rats (n = 7-8 pooled animals per group). All analysis was performed using custom Python scripts. First, data were merged into a single AnnData object with disease status/strain annotation using the Scanpy package[^12^](#_ENREF_12). To ensure the inclusion of only high-quality cells for downstream analysis, cells with fewer than 2100 Unique Molecular Identifiers or expressing less than 500 or more than 3500 genes were excluded to remove low-quality or potential doublet cells, and those with over 15% mitochondrial gene expression were also discarded. Raw counts were converted to log counts per 10,000 by log-normalization and subsequently scaled. To focus on the most informative features, we identified highly variable genes based on specified mean expression and dispersion thresholds (minimum mean of 0.0125, maximum mean of 3, and minimum dispersion of 0.5). After filtering, we corrected for confounding factors by regressing out effects attributable to total counts and mitochondrial gene expression percentages, scaled the data, performed Principal Component Analysis (PCA) for dimensionality reduction, and embed the cells in a two-dimensional space using the Uniform Manifold Approximation and Projection (UMAP) technique.

Cell clusters were identified using the Leiden algorithm (resolution parameter = 0.5). To identify specific cell types, we used Over-Representation Analysis against the PanlaoDB markers for canonical cell types. The technique identified a total of 12171 cells (10545 smooth muscle cells, 844 fibroblasts, 766 endothelial cells, and 16 macrophages). To verify the cell-type annotations, we constructed dot plots for selected marker genes of endothelial cells (vWF, CDH5, VCAM1) smooth muscle cells (CNN1, MYH11, ACTA2), fibroblasts (COL1A1, COL3A1, DCN), and macrophages (LGALS3, CCL3, IL1B). To assess for alterations in the expression of specific ion channels attractable to genetic variances, we subset the data and performed differential expression analysis using a t-test, grouped by animal strain.

REFERENCES

1. Salaun C, Tomkinson NCO, Chamberlain LH. The endoplasmic reticulum-localized enzyme zDHHC6 mediates S-acylation of short transmembrane constructs from multiple type I and II membrane proteins. *J Biol Chem*. 2023;299:105201. doi: 10.1016/j.jbc.2023.105201

2. Wilson C, Zhang X, Buckley C, Heathcote HR, Lee MD, McCarron JG. Increased Vascular Contractility in Hypertension Results From Impaired Endothelial Calcium Signaling. *Hypertension*. 2019;74:1200-1214. doi: 10.1161/HYPERTENSIONAHA.119.13791

3. Wilson C, Lee MD, Buckley C, Zhang X, McCarron JG. Mitochondrial ATP Production is Required for Endothelial Cell Control of Vascular Tone. *Function (Oxf)*. 2023;4:zqac063. doi: 10.1093/function/zqac063

4. Lawton PF, Lee MD, Saunter CD, Girkin JM, McCarron JG, Wilson C. VasoTracker, a Low-Cost and Open Source Pressure Myograph System for Vascular Physiology. *Front Physiol*. 2019;10:99. doi: 10.3389/fphys.2019.00099

5. Heathcote HR, Lee MD, Zhang X, Saunter CD, Wilson C, McCarron JG. Endothelial TRPV4 channels modulate vascular tone by Ca(2+) -induced Ca(2+) release at inositol 1,4,5-trisphosphate receptors. *Br J Pharmacol*. 2019;176:3297-3317. doi: 10.1111/bph.14762

6. Edelstein A, Amodaj N, Hoover K, Vale R, Stuurman N. Computer control of microscopes using microManager. *Curr Protoc Mol Biol*. 2010;Chapter 14:Unit14 20. doi: 10.1002/0471142727.mb1420s92

7. Buckley C, Wilson C, McCarron JG. FK506 regulates Ca(2+) release evoked by inositol 1,4,5-trisphosphate independently of FK-binding protein in endothelial cells. *Br J Pharmacol*. 2020;177:1131-1149. doi: 10.1111/bph.14905

8. McCarron JG, Flynn ER, Bradley KN, Muir TC. Two Ca2+ entry pathways mediate InsP3-sensitive store refilling in guinea-pig colonic smooth muscle. *J Physiol*. 2000;525 Pt 1:113-124. doi: 10.1111/j.1469-7793.2000.00113.x

9. Wilson C, Saunter CD, Girkin JM, McCarron JG. Advancing Age Decreases Pressure-Sensitive Modulation of Calcium Signaling in the Endothelium of Intact and Pressurized Arteries. *J Vasc Res*. 2016;53:358-369. doi: 10.1159/000454811

10. Wilson C, Zhang X, Lee MD, MacDonald M, Heathcote HR, Alorfi NMN, Buckley C, Dolan S, McCarron JG. Disrupted endothelial cell heterogeneity and network organization impair vascular function in prediabetic obesity. *Metabolism*. 2020;111:154340. doi: 10.1016/j.metabol.2020.154340

11. Cheng J, Gu W, Lan T, Deng J, Ni Z, Zhang Z, Hu Y, Sun X, Yang Y, Xu Q. Single-cell RNA sequencing reveals cell type- and artery type-specific vascular remodelling in male spontaneously hypertensive rats. *Cardiovasc Res*. 2021;117:1202-1216. doi: 10.1093/cvr/cvaa164

12. Wolf FA, Angerer P, Theis FJ. SCANPY: large-scale single-cell gene expression data analysis. *Genome Biol*. 2018;19:15. doi: 10.1186/s13059-017-1382-0

**Table S1: Differential Expression Analysis of IP_3_ receptor and TRPV ion channels in Endothelial Cells from mesenteric arteries isolated from WKY and SHR rats.**

| Gene | Score | Log Fold Change | P-Value | Adjusted P-Value |
| --- | --- | --- | --- | --- |
| ITPR1 | -6.1 | -1.3 | 2.2E-09 | 0.000 |
| ITPR2 | -0.7 | -0.4 | 5.0E-01 | 1.000 |
| ITPR3 | -2.8 | -1.4 | 5.0E-03 | 0.023 |
| TRPV1 | 0.0 | 0.0 | 1.0E+00 | 1.000 |
| TRPV2 | -0.3 | -0.7 | 7.6E-01 | 1.000 |
| TRPV3 | 0.0 | 0.0 | 1.0E+00 | 1.000 |
| TRPV4 | 2.6 | 0.6 | 1.1E-02 | 0.032 |
| TRPV5 | 0.0 | 0.0 | 1.0E+00 | 1.000 |
| TRPV6 | 0.0 | 0.0 | 1.0E+00 | 1.000 |

**Table S2: Differential Expression Analysis of IP_3_R and TRPV ion channels in smooth muscle cells from mesenteric arteries isolated from WKY and SHR rats.**

| Gene | Score | LogFoldChange | P-Value | Adjusted P-Value |
| --- | --- | --- | --- | --- |
| ITPR1 | -45.7 | -2.6 | 0.0E+00 | 0.000 |
| ITPR2 | 1.4 | 18.7 | 1.6E-01 | 0.472 |
| ITPR3 | -0.6 | -1.2 | 5.2E-01 | 0.891 |
| TRPV1 | -0.5 | -0.4 | 5.9E-01 | 0.891 |
| TRPV2 | 2.1 | 0.4 | 3.5E-02 | 0.157 |
| TRPV3 | 0.0 | 0.0 | 1.0E+00 | 1.000 |
| TRPV4 | -0.7 | -0.3 | 5.1E-01 | 0.891 |
| TRPV5 | 0.0 | 0.0 | 1.0E+00 | 1.000 |
| TRPV6 | 0.0 | 0.0 | 1.0E+00 | 1.000 |

**SUPPLEMENTARY FIGURES**


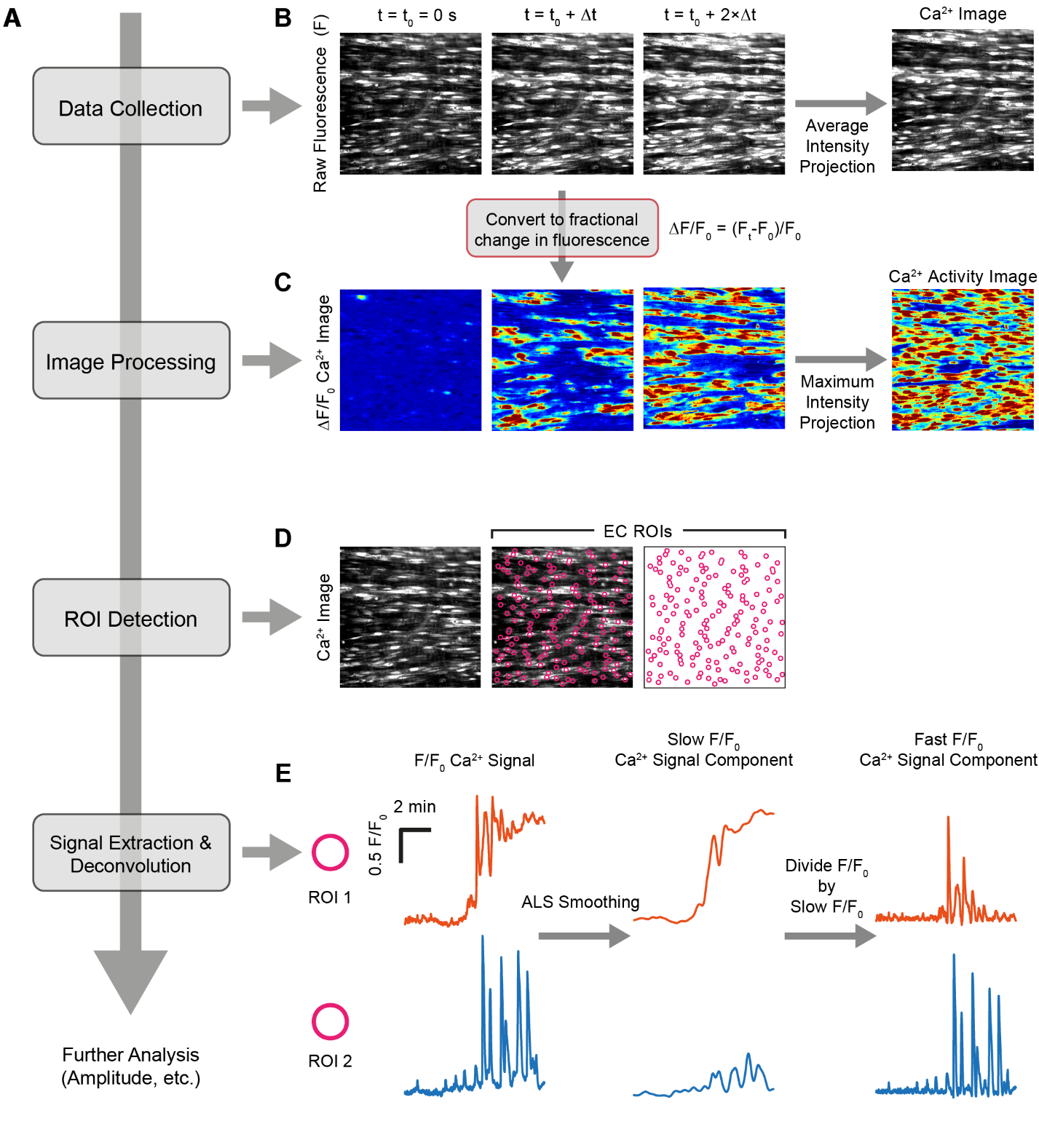


**Figure S1 – Visualizing Ca^2+^ activity and extracting single cell Ca^2+^ signals**. (A-E) Sequential illustration of processing steps to summarize Ca^2+^ imaging data. (B) Data collection: fluorescence images are collected over time to create an image stack representing Ca^2+^ activity. This set of three-dimensional data (xyt) can be condensed into a single, representative Ca^2+^ image by averaging all frames within the image stack. (C) Image processing: the raw images from (B) are processed to generate images representing the fractional increase in fluorescence intensity over baseline (ΔF/F_0_), where F_0_ is the baseline fluorescence and ΔF is baseline-corrected fluorescence at time t (F_t_-F_0_). This dataset can be condensed into a single, “Ca^2+^ activity image” by taking an average or maximum intensity projection. (D) ROI Detection: circular regions of interest (ROI) are generated semi-automatically using custom software (see Methods). All cells in the field are assigned a single ROI. (E) Signal extraction and deconvolution: The fluorescence signal from each ROI (cell) is extracted and converted to the baseline-corrected fluorescence ratio, F/F_0_. These signals are then smoothed using an asymmetric least squared fit to remove all oscillatory transient events (middle panel), thereby isolating the slow sustained component of the Ca^2+^ signal. In the right-hand panel, the slow component is removed from the raw F/F_0_ signal (left panel) by frame-by-frame division. This process reveals the transient oscillatory fast component of the Ca^2+^ signal.


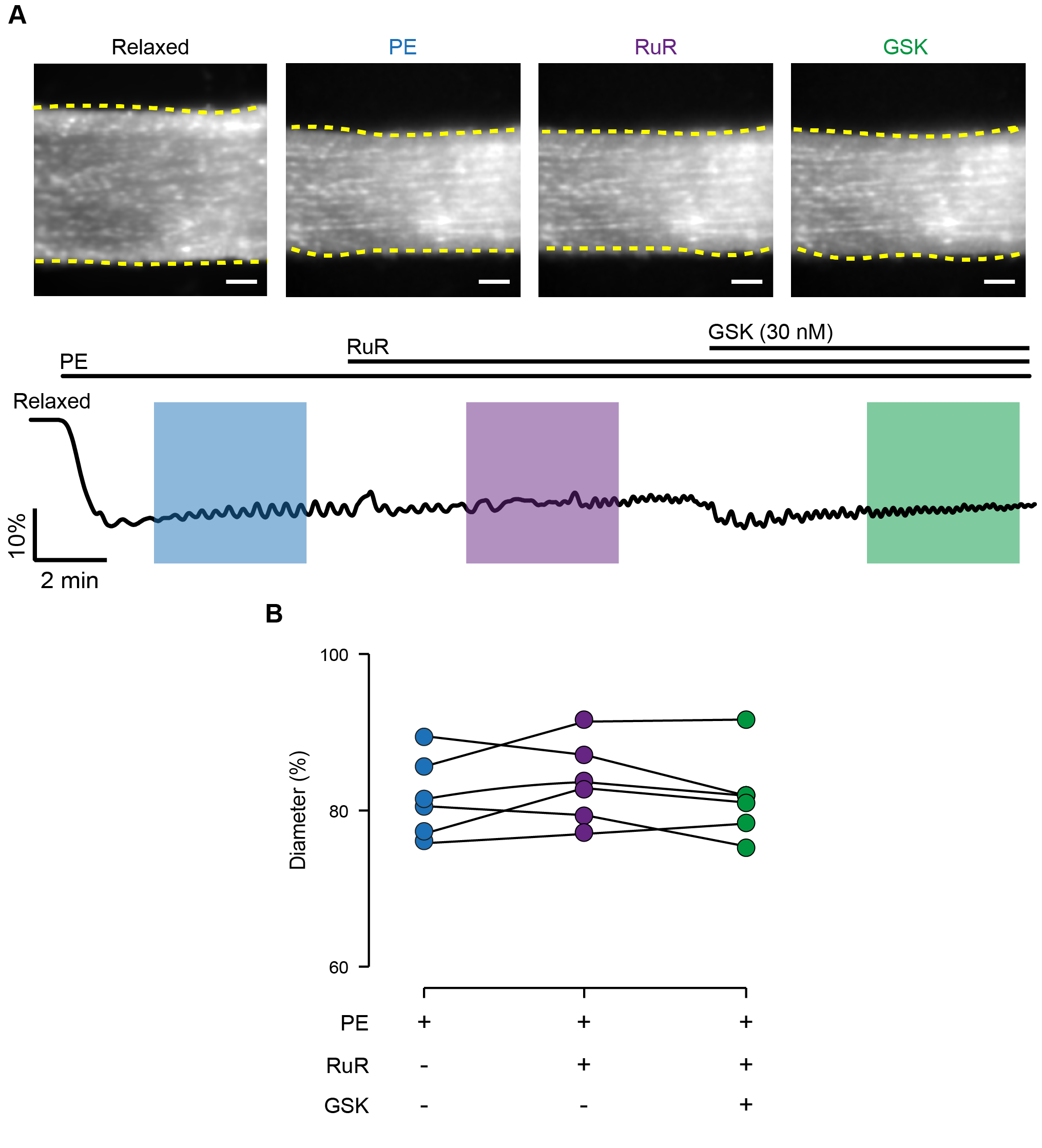


**Figure S2 – TRPV4 inhibition does not reverse PE-induced contraction.** A-B) Still frame images/time course (A) and summary data (B) showing the effect of the TRP channel inhibitor, ruthenium red (5 μM), and the selective TRPV4 channel agonist, GSK1016790A (GSK, 30 nM), on mesenteric artery tone during vasoconstriction evoked by phenylephrine (PE, 2 μM). Data were assessed using a one-way ANOVA for paired data with Tukey’s post-hoc test for multiple comparisons. Image scale bars = 100 µm (n = 6).


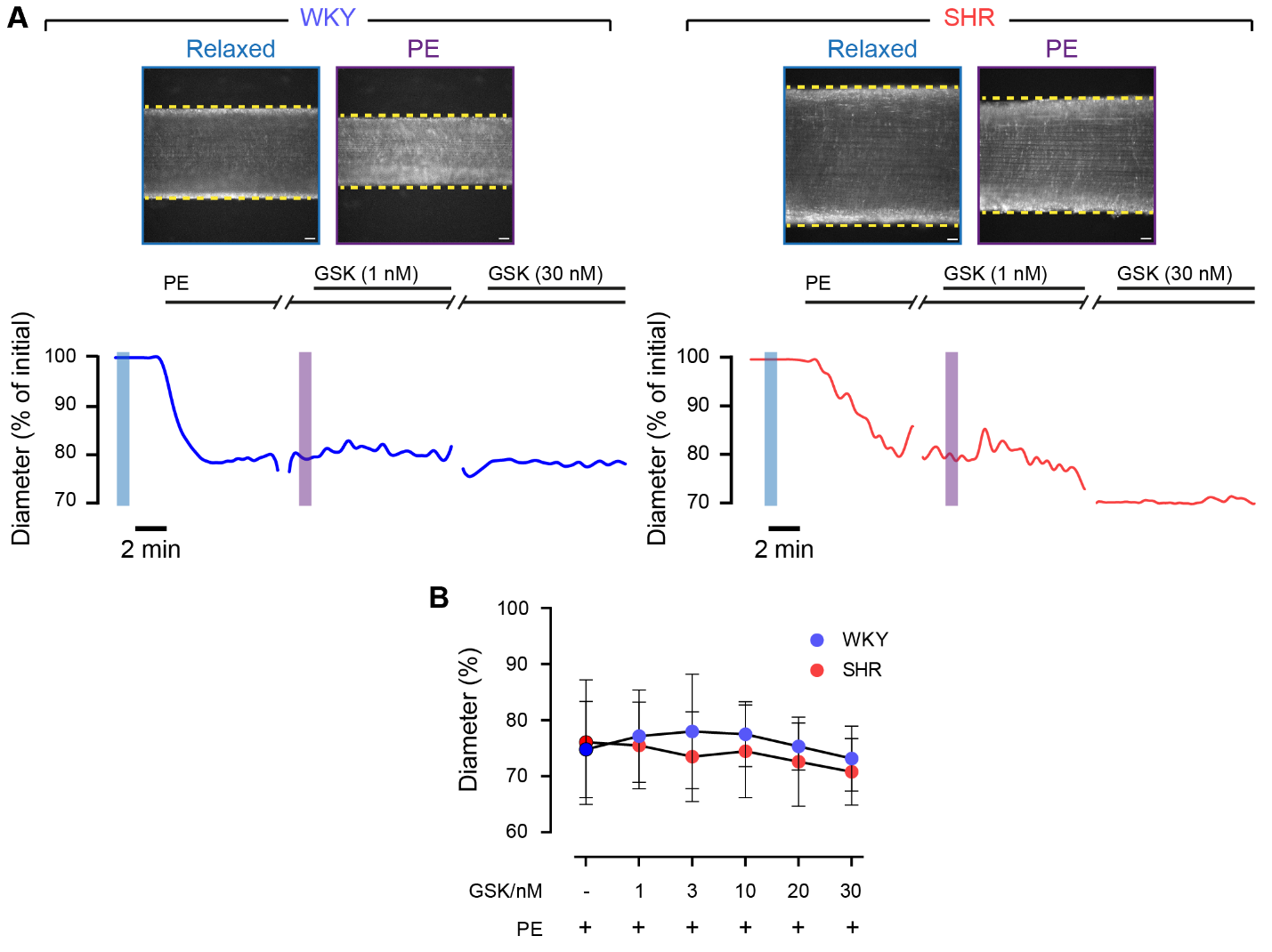


**Figure S3 – TRPV4-mediated regulation of vascular tone is endothelial dependent.** A-B) Still frame images/time course (A) and summary data (B) showing, in the absence of the endothelium, the effect of the TRPV4 channel agonist GSK1016790A (GSK) on mesenteric artery tone during vasoconstriction evoked by phenylephrine (PE, 2 μM). The lower panel shows diameter traces of the arteries, measured using Vasotracker, at rest, after PE and after GSK. The colored lines correspond to the timepoints shown in the images above. Endothelial cells were mechanically removed with a fine hair, and these arteries remained capable of constricting to phenylephrine (PE) and relaxing to the nitric oxide donor, sodium nitroprusside (SNP; see Figure S4). Image scale bars = 50 µm.


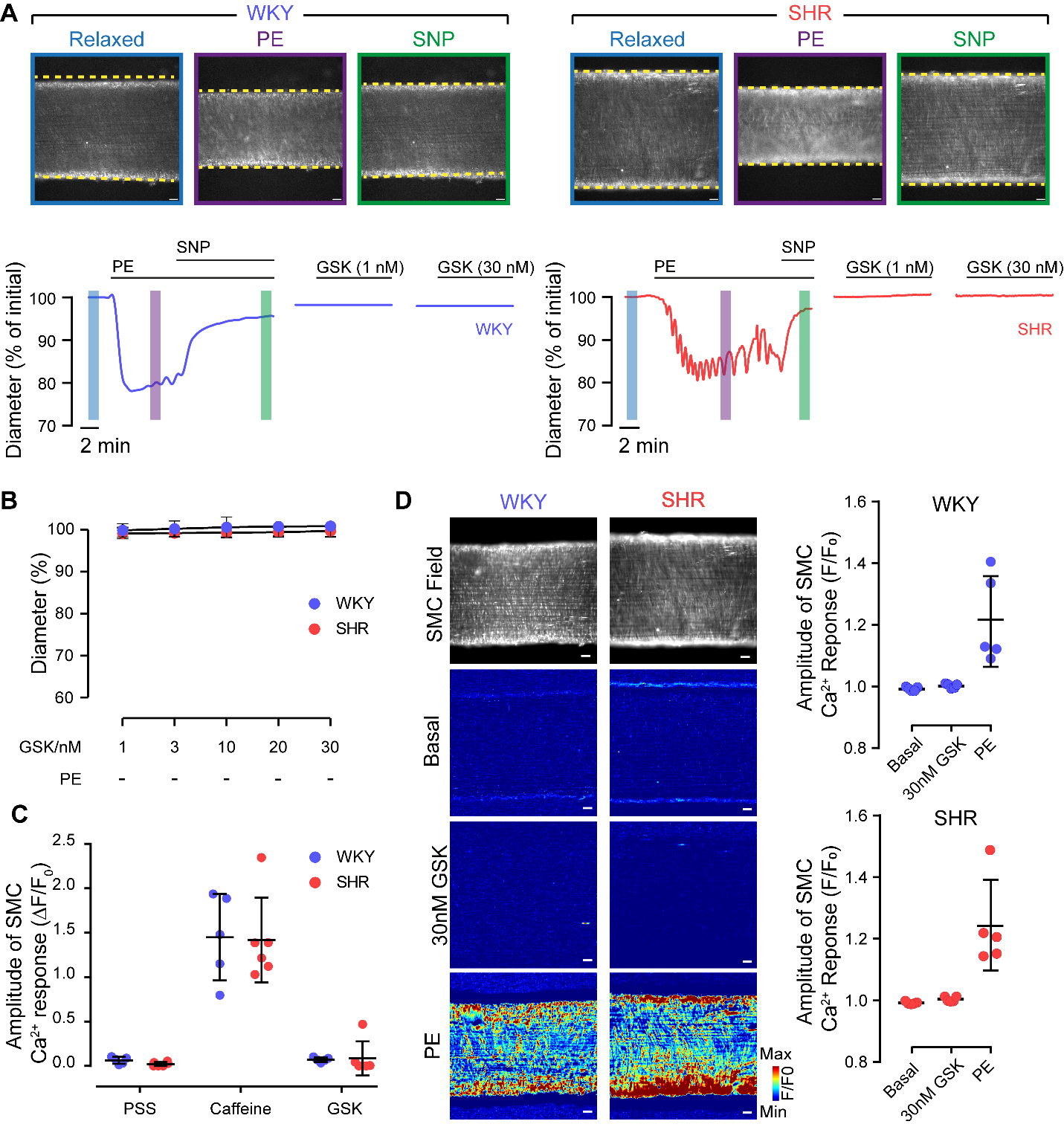


**Figure S4 – TRPV4-mediated vasoconstriction is absent in endothelium-denuded arteries and TRPV4 appears non-functional in smooth muscle cells.** (A) Still frame images and diameter traces showing the effect of phenylephrine (PE) and sodium nitroprusside (SNP) on vascular tone in mesenteric arteries from WKY (left) and SHR (right) in which the endothelial cell layer had been mechanically removed with a fine hair. The diameter traces in the bottom row illustrate the full time course of the experiments, and were measured using Vasotracker. The colored lines correspond to the timepoints shown in the top row. Although arteries contracted to PE, and dilated to SNP, they did not contract to GSK. (B) Summarized (mean ± SEM; n=6) data showing the response of the arteries from WKY and SHR animals to increasing concentration of GSK in the absence of PE. (C) Summary data (mean ± SD) showing the amplitude of agonist-evoked Ca^2+^ responses in isolated single smooth muscle cells from mesenteric arteries from WKY and SHR animals. The responses of smooth muscle cells to PSS (control), caffeine (10 mM) and GSK (30 nM) are shown. (D) Raw images, maximum intensity projection (i.e. total Ca^2+^ responses) and summarized data in intact arteries from WKY and SHR animals after endothelium removal, showing that GSK (30 nM) did not increase smooth muscle Ca^2+^ concentration, while PE was effective in increasing smooth muscle Ca^2+^ concentration (n=5). Image scale bars = 50 µm.


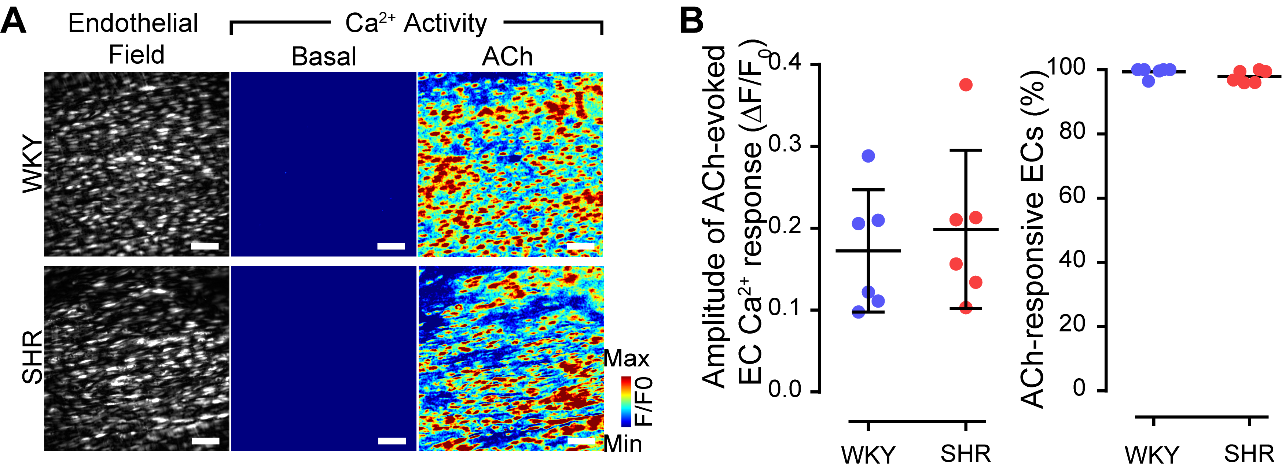


**Figure S5 Acetylcholine-evoked endothelial responses are maintained in hypertension.** A) Endothelial cell Ca^2+^ responses to ACh (50 nM) in arteries from WKY and SHR animals. Images show the field of endothelial cells (left) and ΔF/F_0_ maximum intensity projections (i.e. total Ca^2+^ responses) at rest (basal), and after ACh. B) Summary data (mean ± SD; n=6) illustrating the amplitude of ACh-evoked Ca^2+^ activity (left) and number of ACh-responsive cells (right) in SHR and WKY animals. Image scale bars = 50 µm.


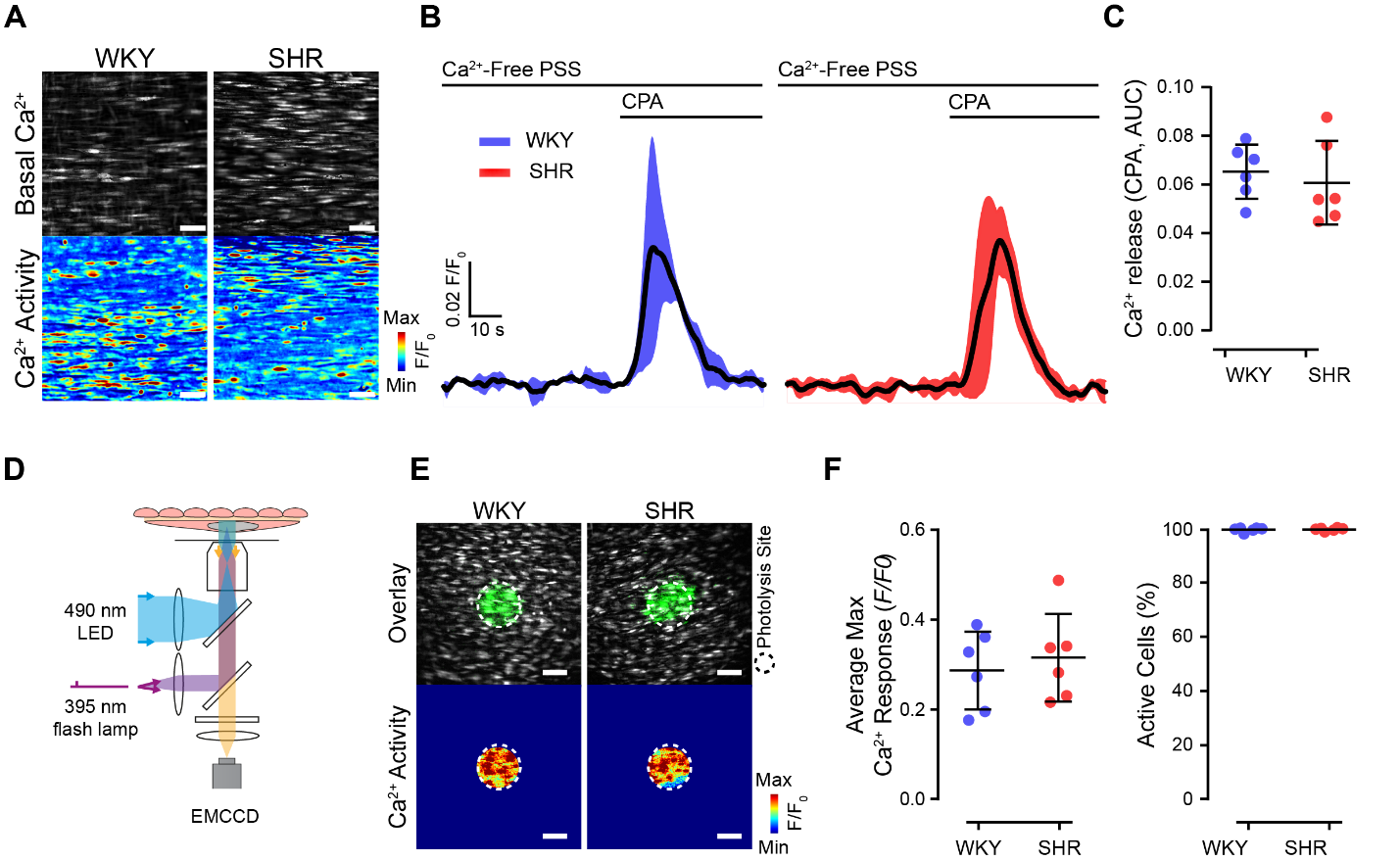


**Figure S6 – IP_3_ receptor activity, and the internal Ca^2+^ store content, are maintained in hypertension.** (A) Top panel: Intact endothelial cell field, lower panel: heatmap of Ca^2+^ signals evoked by the Ca^2+^ pump inhibitor, cyclopiazonic acid (CPA, 20 µM) in the same preparations shown in the top panels. (B) Averaged Ca^2+^ transients evoked by depletion of the internal store by cyclopiazonic acid (CPA; 20 µM). CPA was applied in a Ca^2+^-free PSS. The black line is the mean and the shaded region shows the standard deviation (n=6). (C) Summary data of the integrated (area under the curve, AUC) Ca^2+^ release from the data shown in (B) from WKY (blue) and SHR (red) animals. Data show mean ± SD. (D) Schematic of imaging system for targeted endothelial cell photoactivation. (E) Example Ca^2+^ activity response within the uncaging region (dotted circle) after photorelease of caged IP_3_. Top panel: Ca^2+^ activity (green) is overlaid onto the endothelial field (grey). Bottom panel: magnitude of Ca^2+^ activity is shown using a heatmap. (F) Mean (± SD) summary data (n = 6) illustrating the peak response to activation of endothelial IP_3_ receptors via photolysis of caged IP_3_ (left panel) and percentage of cells activated (right panel) within the uncaging region. Statistical significance (P < 0.05) was assessed using a Student’s t-test. Image scale bars = 50 µm.


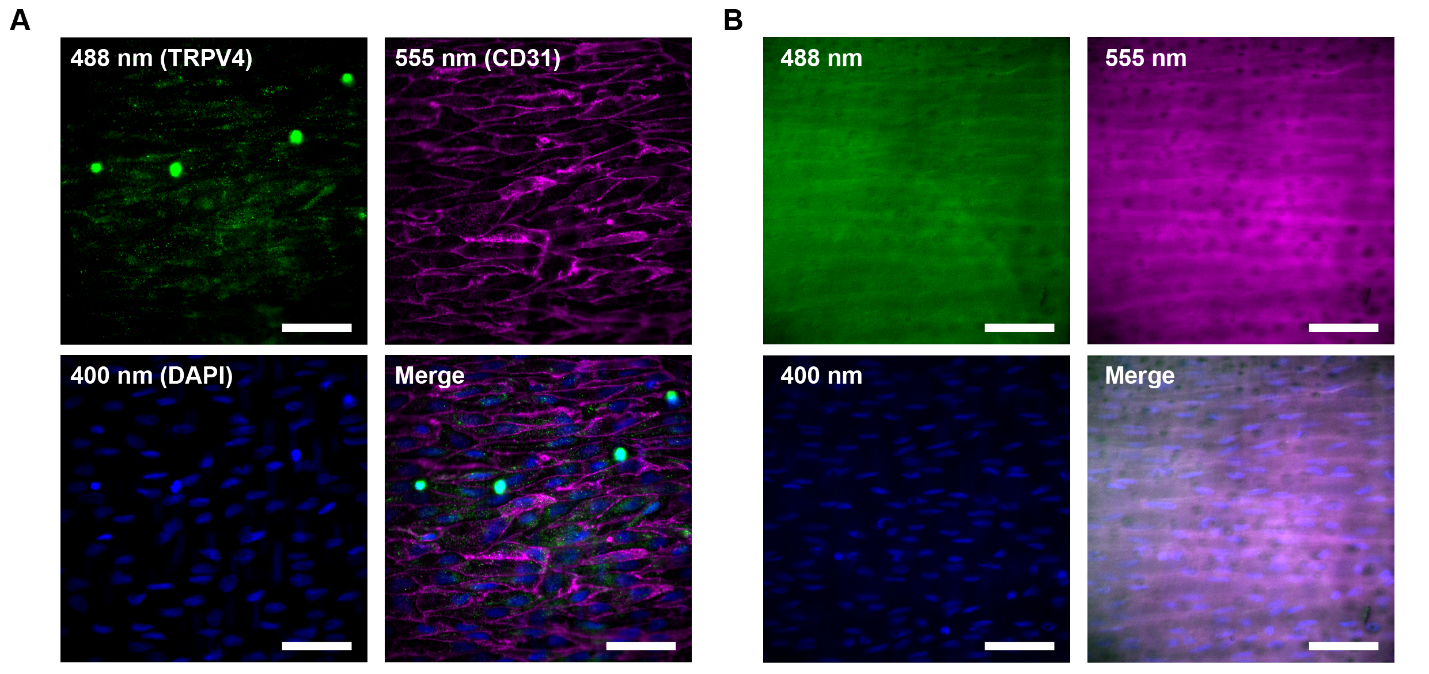


**Figure S7– Immunofluorescence staining of endothelial cells in intact arteries.** A) Endothelial cell TRPV4 ion channel expression revealed by in situ fluorescence imaging. Images show specific labelling of endothelial cells using primary antibodies against the target antigens: TRPV4 receptor (green), cell-cell borders (CD31, magenta) and nuclei (DAPI, blue). B) Representative images from control experiments with no primary antibody. No fluorescence signal was detected in arteries incubated with secondary antibodies alone. Scale bars = 50 µm.


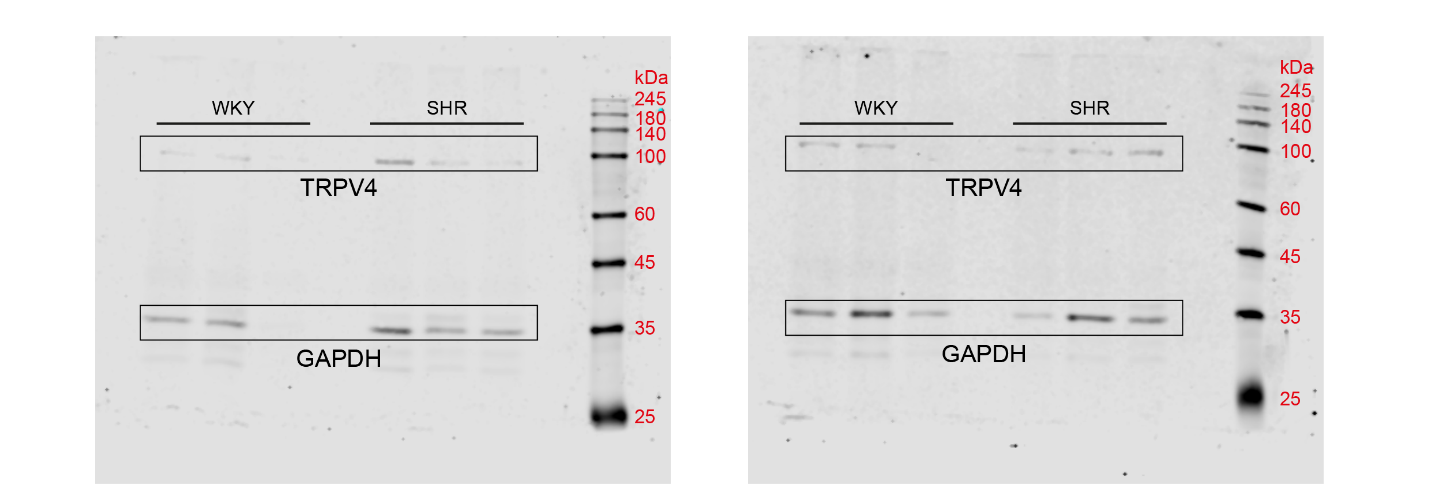


Figure S8. TRPV4 protein expression is upregulated in arteries from hypertensive animals. Equal loading of total protein (30 µg) from mesenteric arteries from WKY and SHR animals (n= 6) were assessed by Western blotting against TRPV4 (97 kDa) and a housekeeping protein, GAPDH (36 kDa).

**Supplementary Movie**

**Supplementary Movie 1 – Endothelial TRPV4 Channel Activation by GSK (30 nM) in arteries from WKY and SHR animals.** TRPV4 channel activation by 30 nM GSK in WKY (left panel) and SHR (right panel) over a 10-minute recording. Time (mm:ss) is indicated on the top right of each panel and an increase in fluorescence reflects increased intracellular Ca^2+^ concentration. There is an increased shift from oscillatory Ca^2+^ changes to sustained responses in hypertension.
